# Supplementary figures and images for: Iterative point set registration for aligning scRNA-seq data
Source: PLoS Comput Biol. 2020 Oct 27;16(10):e1007939. doi: 10.1371/journal.pcbi.1007939 (PMC7647120; doi:10.1371/journal.pcbi.1007939)

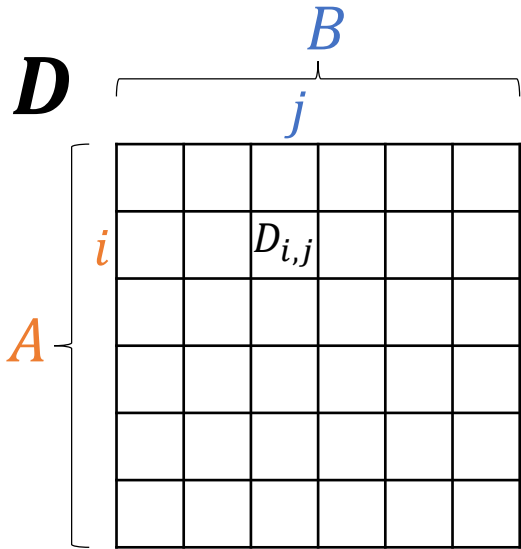

Supplement: S1 Fig — D ∈ ℝn×m where n = |A| and m = |B| and Di,j is the distance between cells Ai and Bj. In our work, A is called the “source” set and B is called the “target” set. We use the euclidean distance throughout our paper. (PDF) [file pcbi.1007939.s001.pdf]

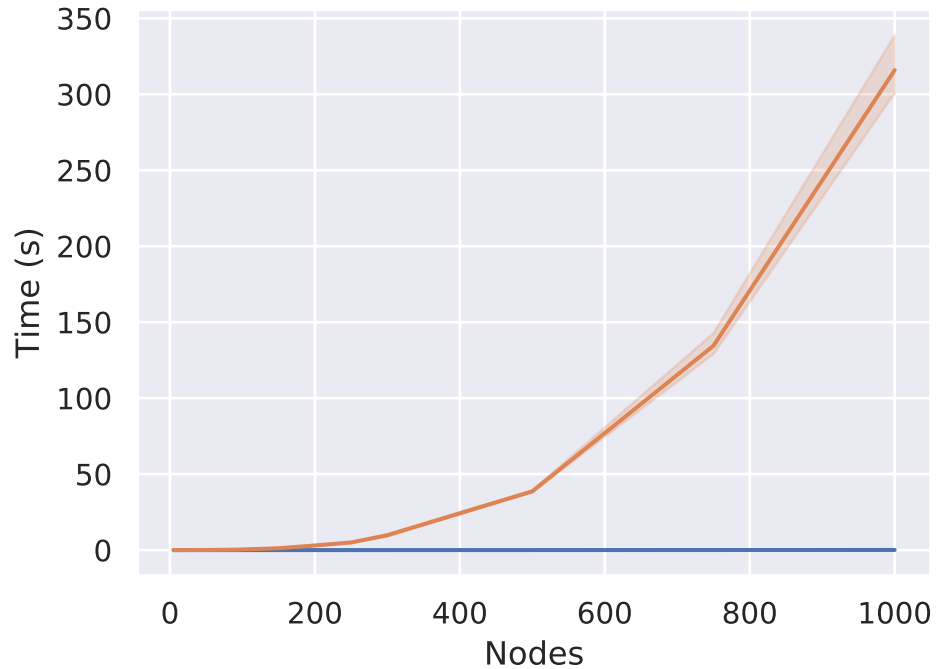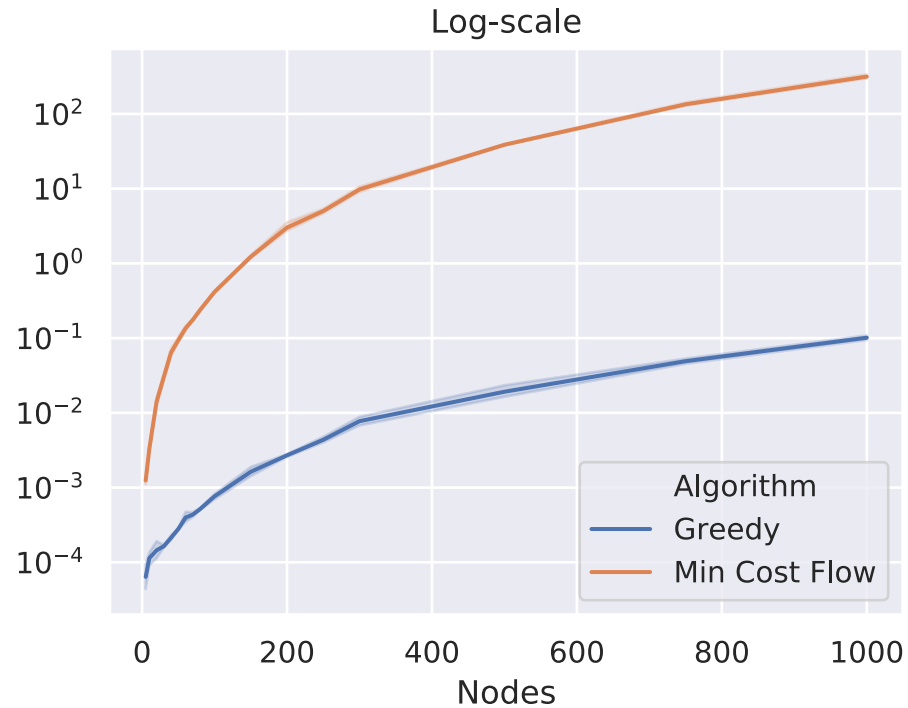

Supplement: S2 Fig — For both algorithms, we generated random distances matrices with varying numbers of cells (also called nodes). In this simple case designed to test algorithm runtimes as a funciton of input size, the distances were integers uniformly drawn from [0, 50]. The distance matrices are square, representing the case when a source batch has the same number of cells as the output batch, where the x-axis in the above plots is the number of cells in each batch. For the greedy algorithm, we used the default parameters as discussed in the main body and in S1 Appendix section “Parameter settings for SCIPR experiments”. For the Min Cost Flow algorithm, we started by constructing a bipartite graph where nodes in one set represented cells in the source batch, and nodes in the other set represented cells in the target set. The directed (from source to target) edge weights (costs) were set to the distances between the nodes as given by the randomly generate distance matrix. Then a “source” node was added and connected to all of the nodes of the source cells, and a “sink” node was added and all of the nodes of the target cells were connected to it. The demand of the source node was set to -0.5 × nodes (the number of “units” of flow that this node wants to send, i.e. the number of pairings of cells we want to assign), and the demand of the sink node was set to negative of this (the number of “units” of flow that it wants to receive). Finally, the capacity of each edge in this directed network was set to 1, and the the network simplex algorithm was used to find a solution. The directed graph was constructed using the NetworkX python package and they network simplex algorithm was run via the min_cost_flow function [43]. (PDF) [file pcbi.1007939.s002.pdf]

CELseq2  $\rightarrow$  10x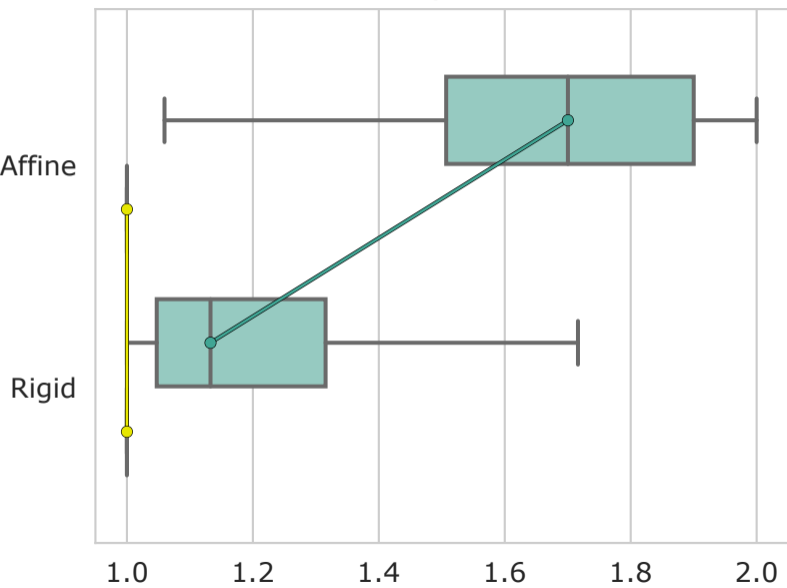Dropseq  $\rightarrow$  10x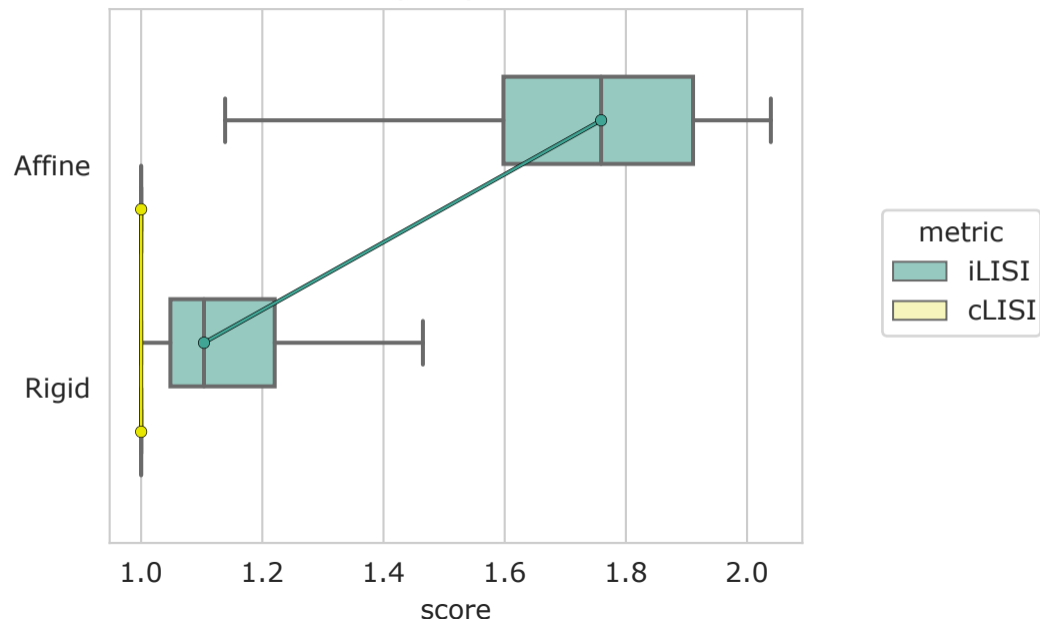

Supplement: S3 Fig — These alignment tasks are from the CellBench dataset, the smallest and somewhat easiest dataset. The two subplots use different source batches (both are aligned to the same largest reference batch, 10x). The scores are iLISI (green, batch integration score), and cLISI (orange, cell type mixing score). In each subplot the methods are ordered from top to bottom in order of largest difference (median iLISI − median cLISI) of scores. The center of each box is the median, and whiskers represent 1.5 times the IQR past the low and high quartiles. Circle markers are placed on the medians and connected between boxes with lines of the corresponding color to facilitate visual comparisons. We can see that even on this rather small dataset, the rigid transformation functions are not sufficient to integrate the data well (low iLISI scores), and we see a large gap in iLISI compared with the affine transformation functions. (PDF) [file pcbi.1007939.s003.pdf]

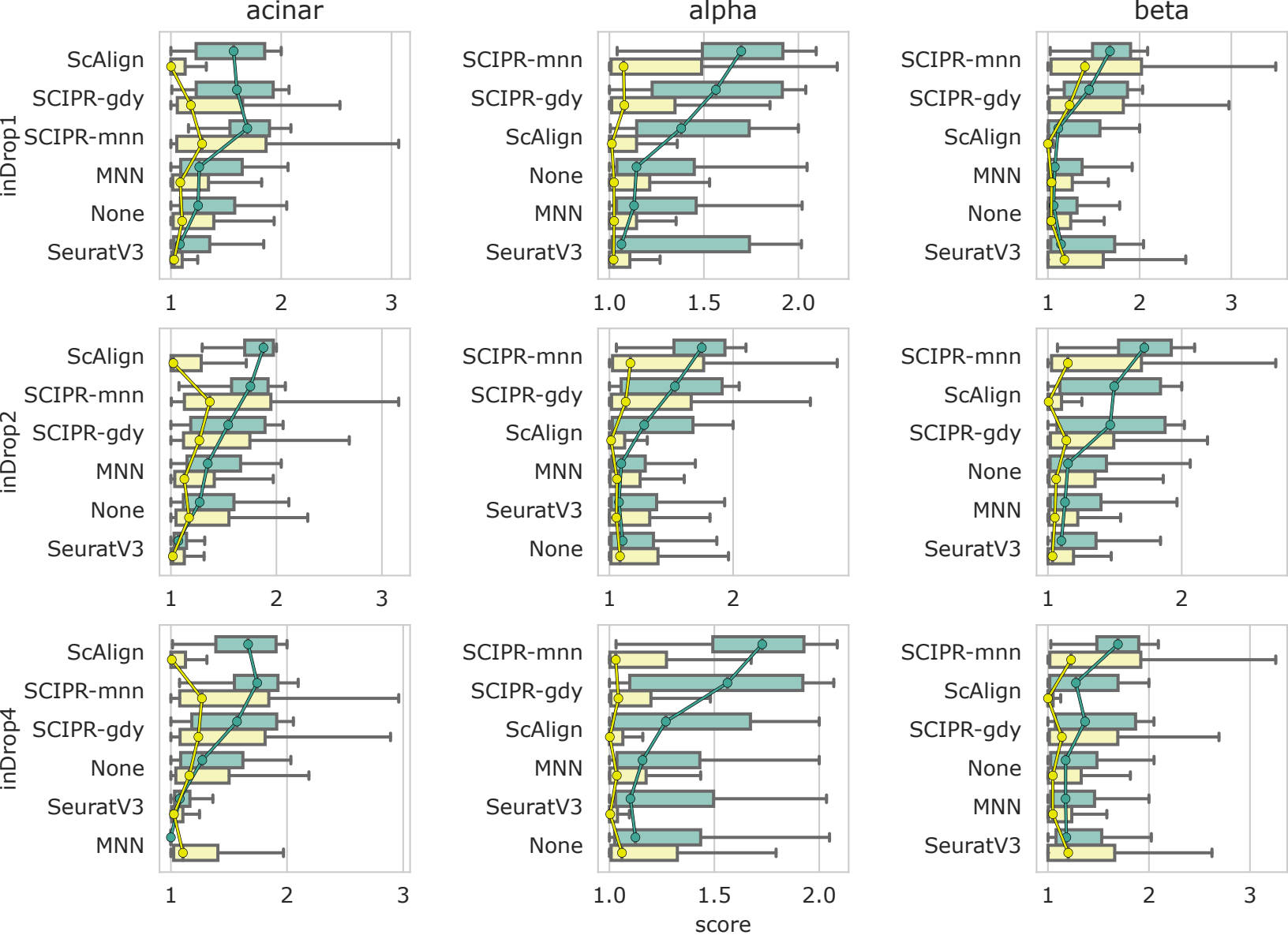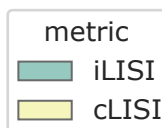

Supplement: S4 Fig — Each row of subplots are alignment tasks with the same source batch, where each column uses a different cell type as a hold-out from the target set (the inDrop3 batch). Box plot computation and ordering of methods in each subplot is determined in the same fashion as in S3 Fig. (PDF) [file pcbi.1007939.s004.pdf]

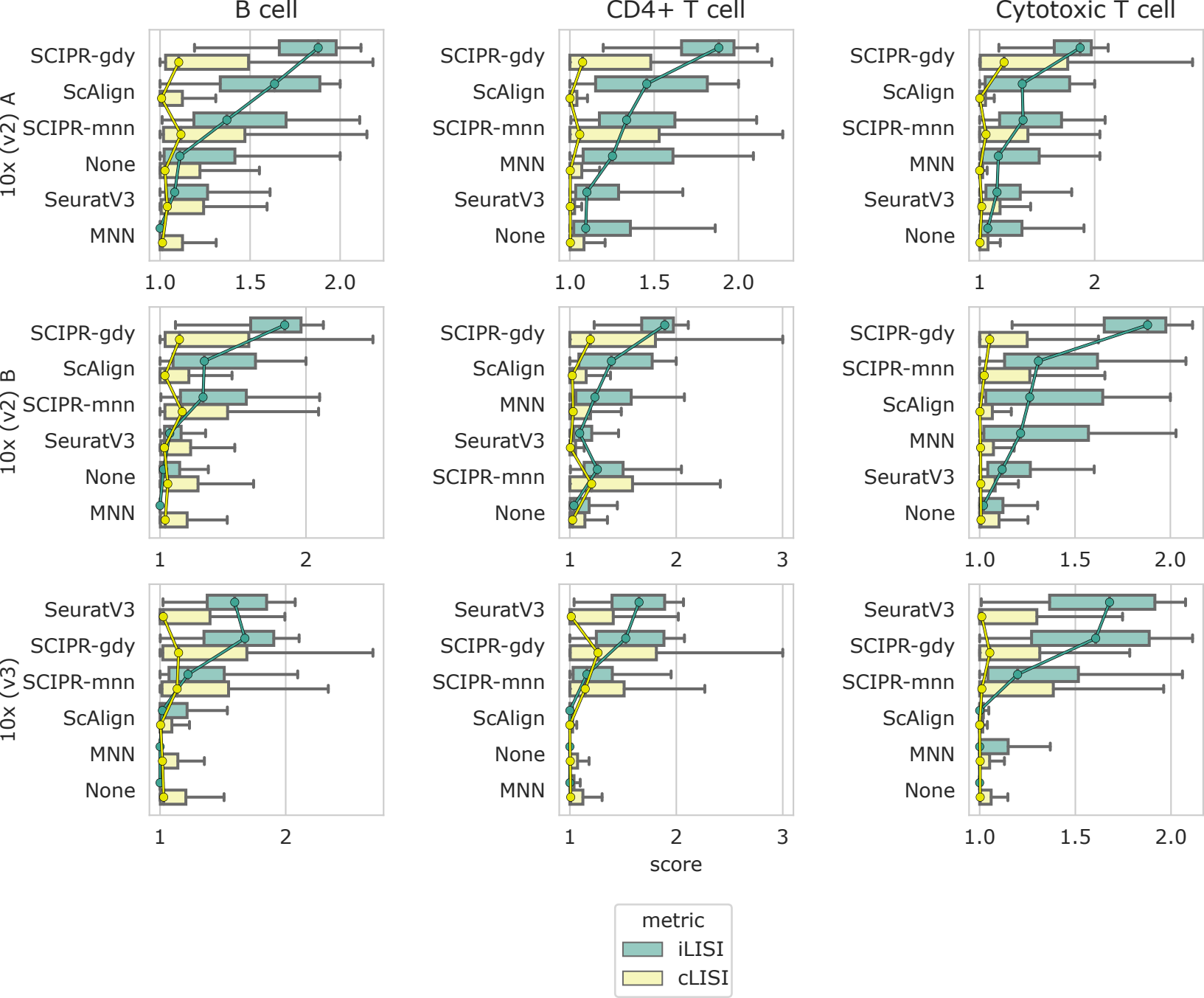

Supplement: S5 Fig — Each row of subplots are alignment tasks with the same source batch, where each column uses a different cell type as a hold-out from the target set (the 10x Chrom. (v2) batch). Box plot computation and ordering of methods in each subplot is determined in the same fashion as in S3 Fig. (PDF) [file pcbi.1007939.s005.pdf]

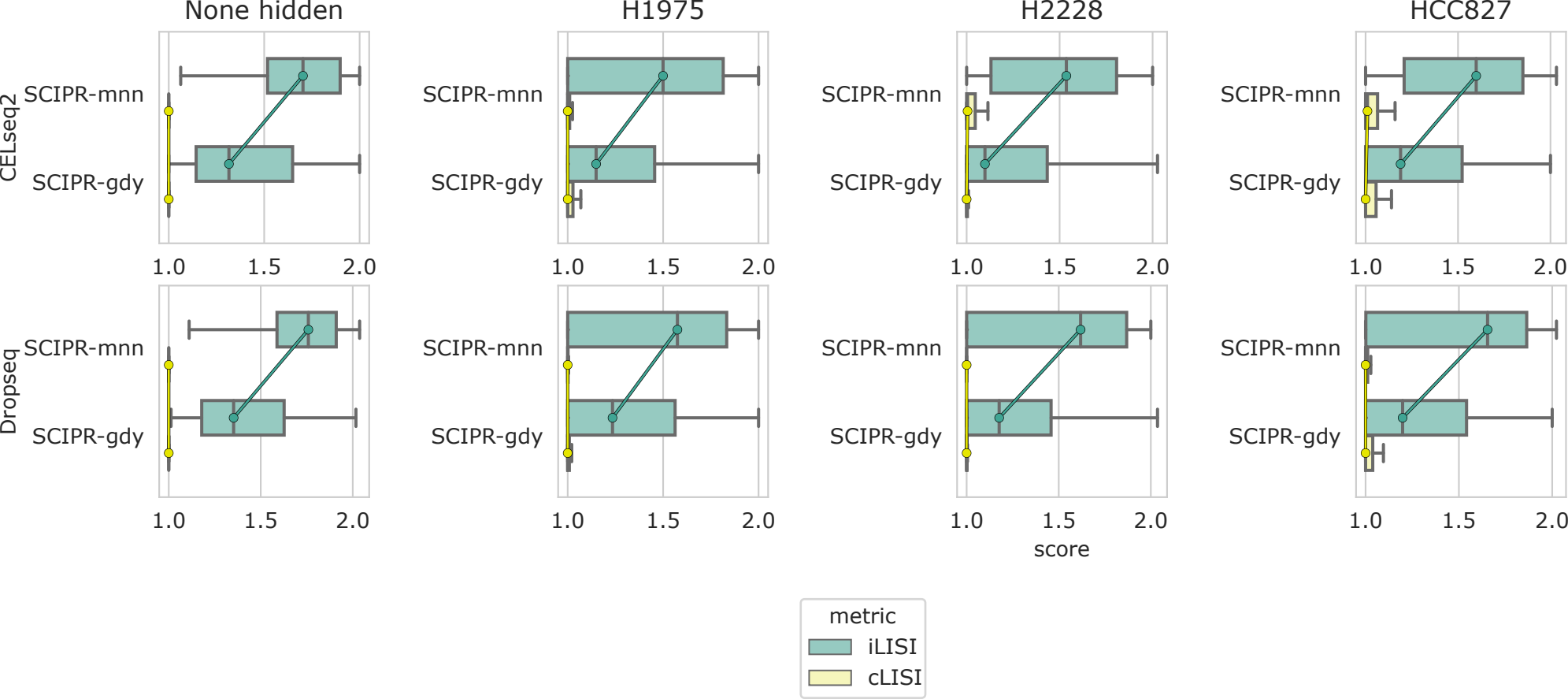

Supplement: S6 Fig — Each row of subplots are alignment tasks with the same source batch, where each column uses a different cell type as a hold-out from the source set. The target set is 10x for all. In the first column, “None hidden”, no cells were hidden from the source set. Box plot computation and ordering of methods in each subplot is determined in the same fashion as in S3 Fig. (PDF) [file pcbi.1007939.s006.pdf]

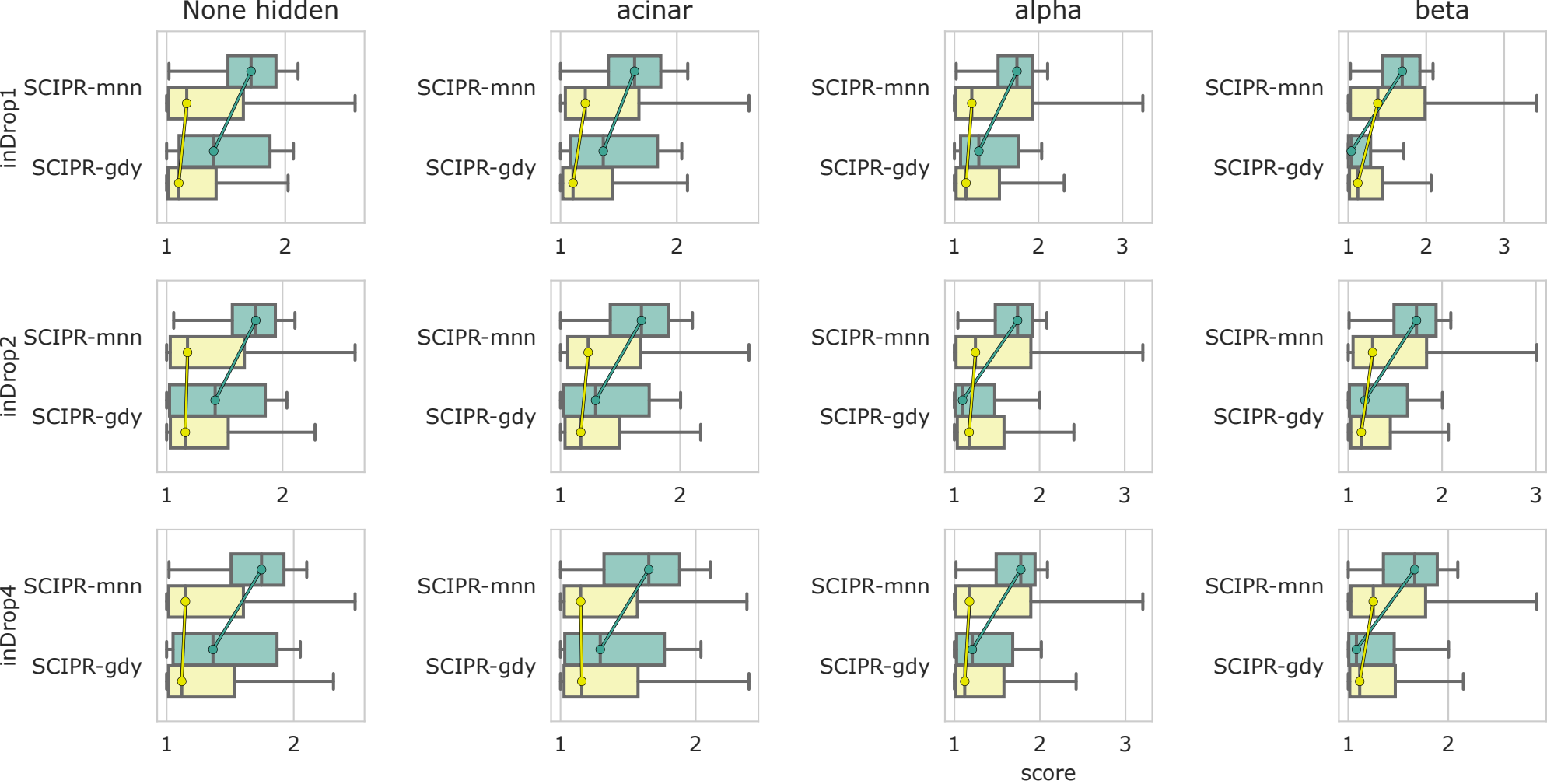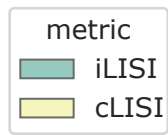

Supplement: S7 Fig — Each row of subplots are alignment tasks with the same source batch, where each column uses a different cell type as a hold-out from the source set. The target set is inDrop3 for all. In the first column, “None hidden”, no cells were hidden from the source set. Box plot computation and ordering of methods in each subplot is determined in the same fashion as in S3 Fig. (PDF) [file pcbi.1007939.s007.pdf]

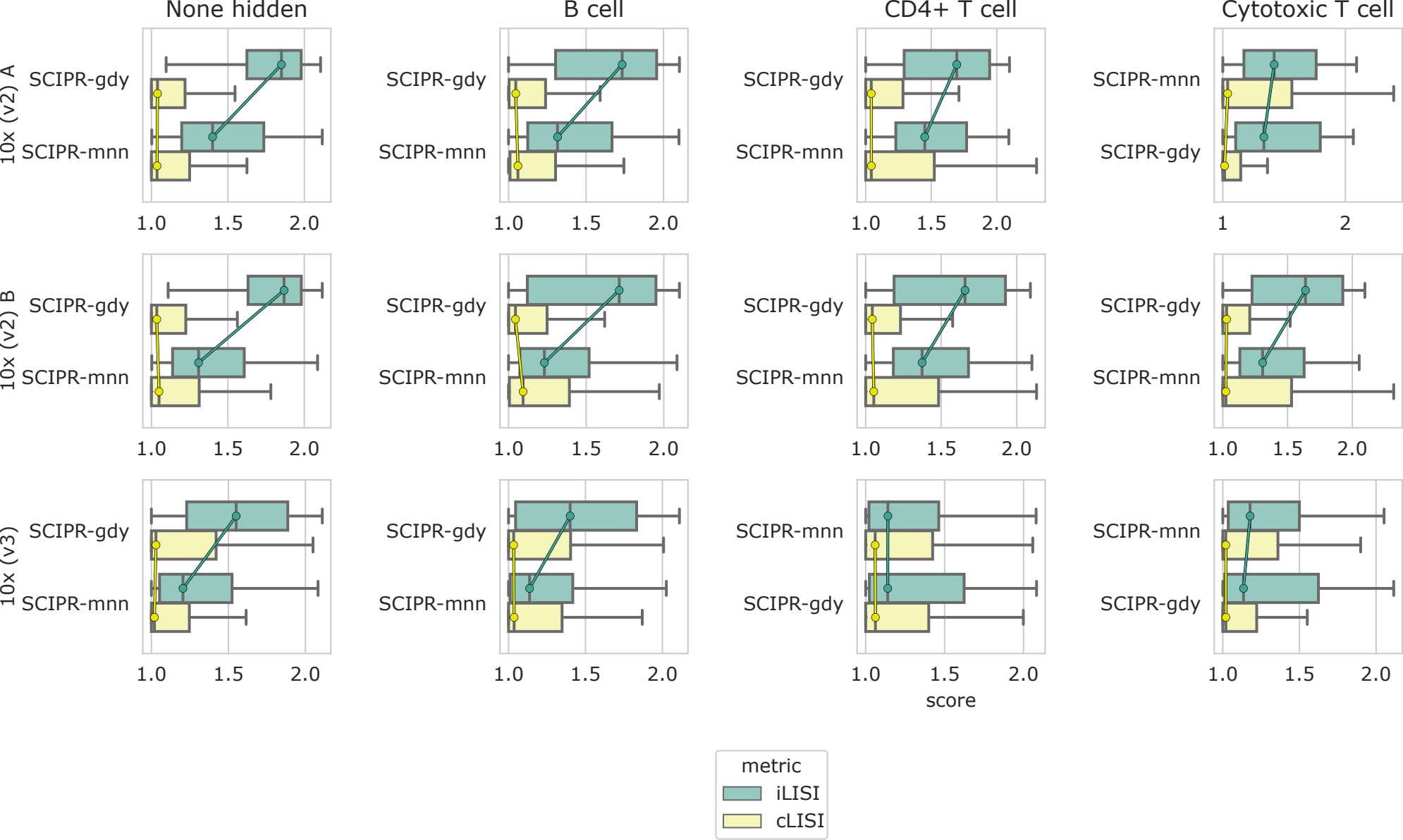

Supplement: S8 Fig — Each row of subplots are alignment tasks with the same source batch, where each column uses a different cell type as a hold-out from the source set. The target set is 10x (v2) for all. In the first column, “None hidden”, no cells were hidden from the source set. Box plot computation and ordering of methods in each subplot is determined in the same fashion as in S3 Fig. (PDF) [file pcbi.1007939.s008.pdf]

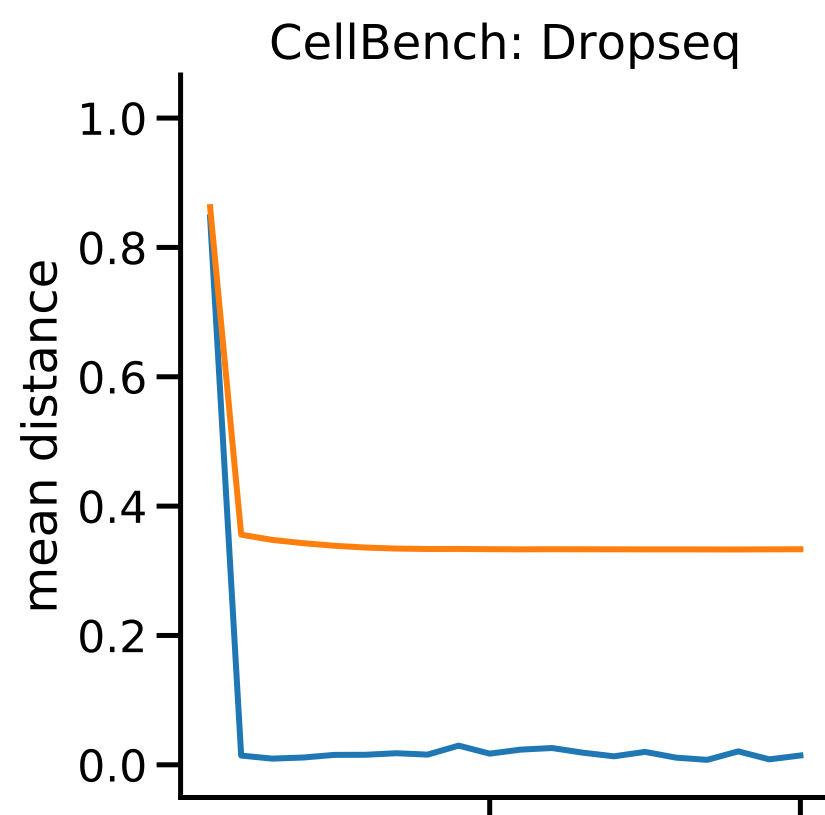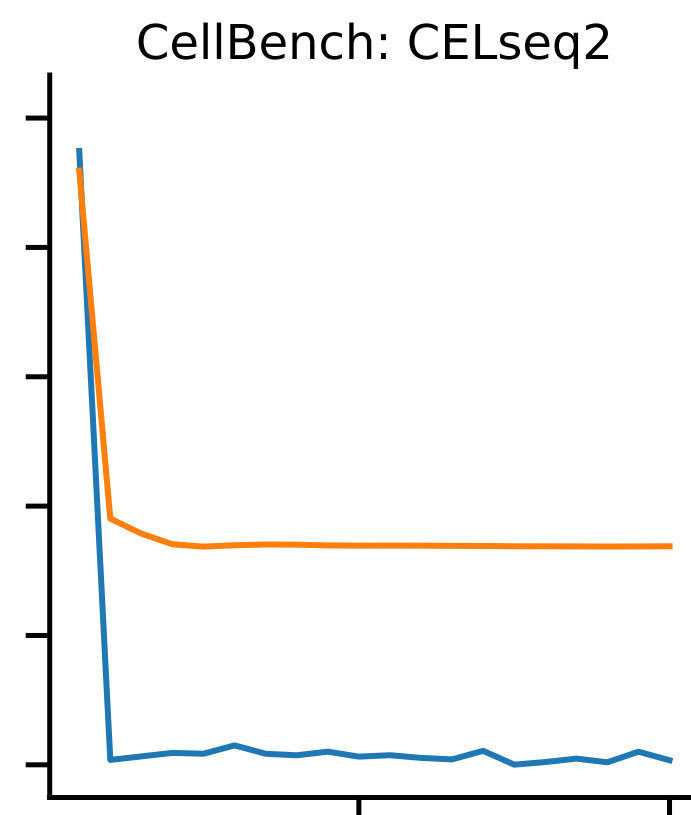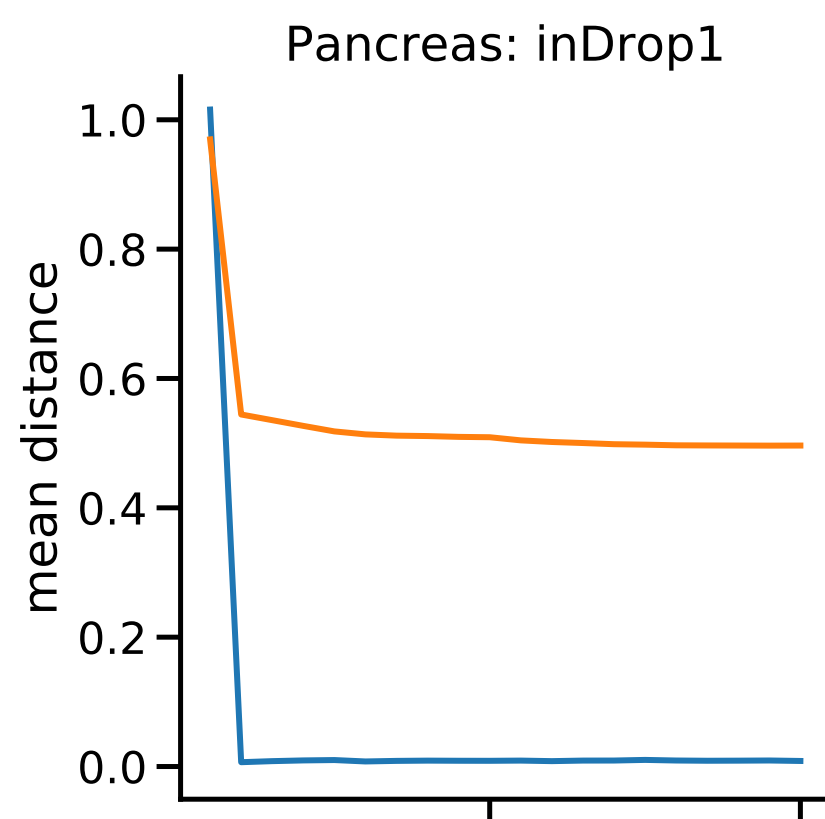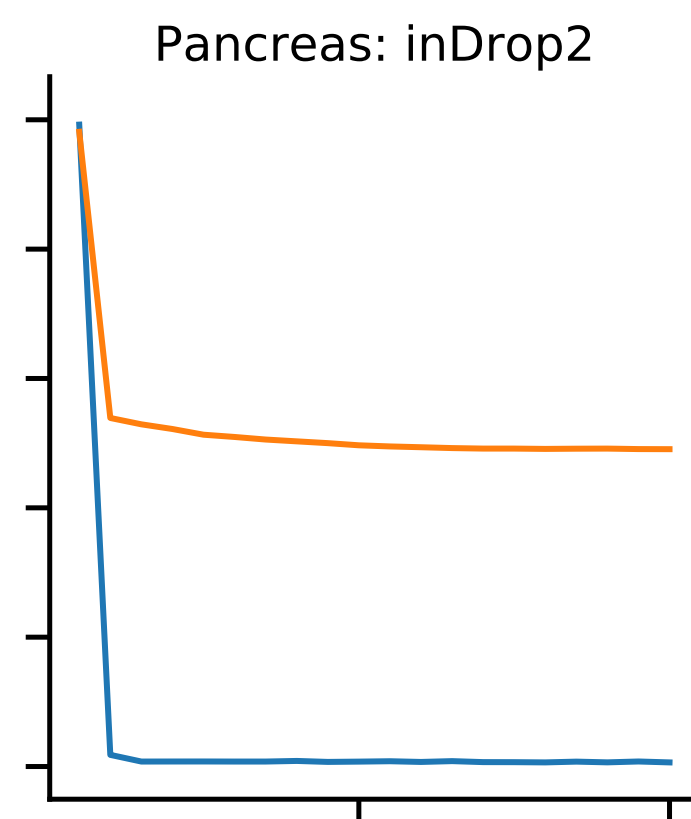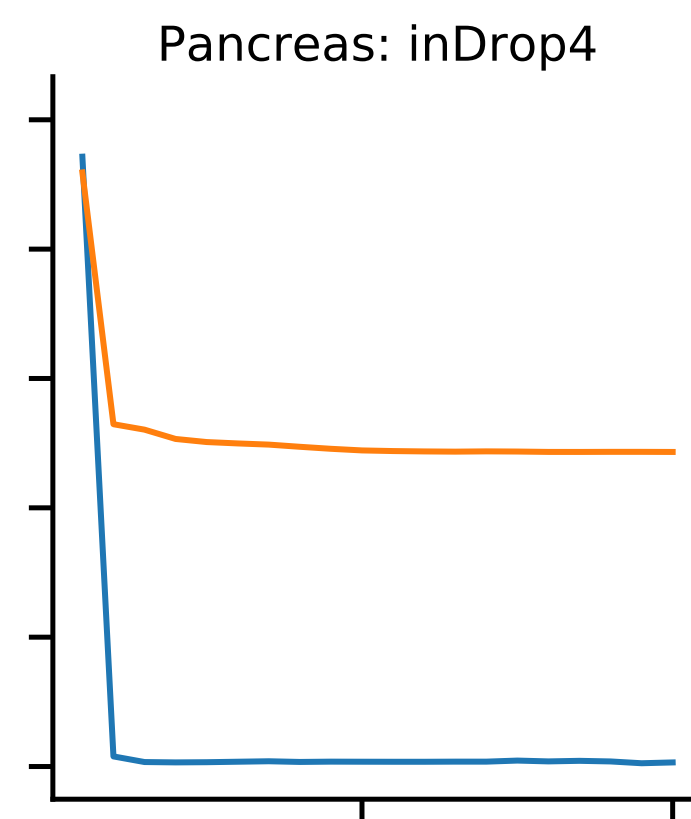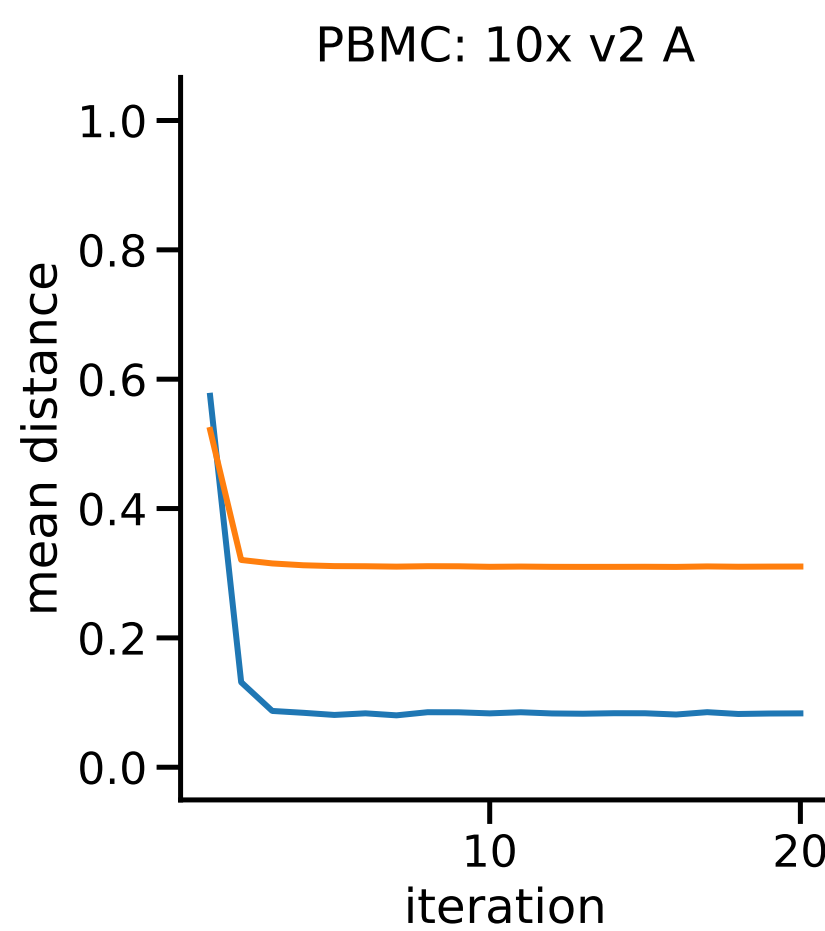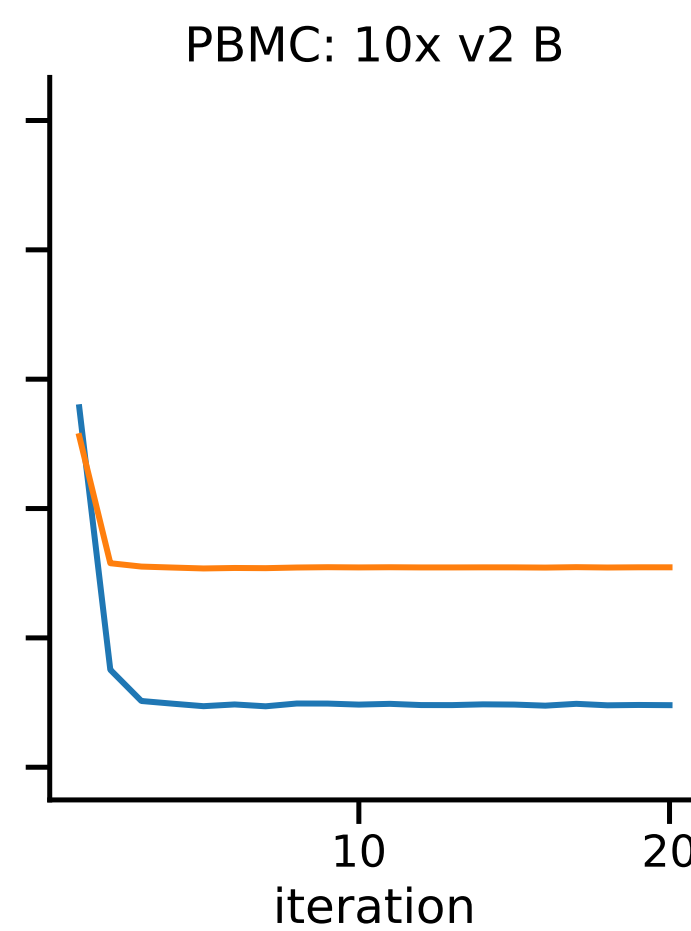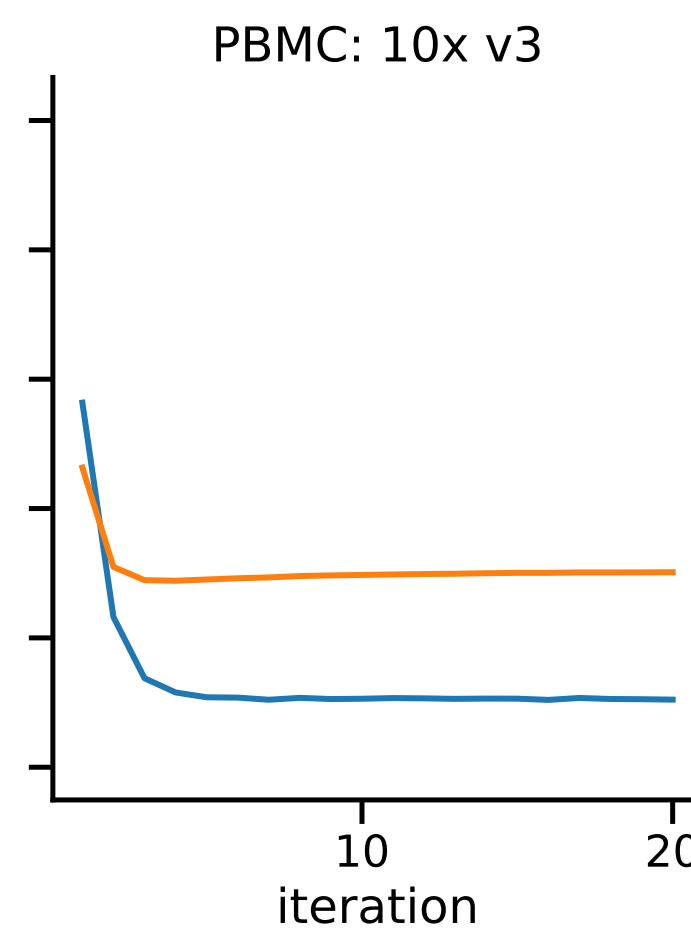

method

- SCIPR-gdy
- SCIPR-mnn

Supplement: S9 Fig — Each row of subplots are tasks from the same dataset, where each column uses a different source batch (all are aligned to the same largest reference batch, 10x for CellBench, inDrop3 for Pancreas, and 10x v2 for PBMC). The values plotted are the mean distances between the selected pairs of points after each iteration of the algorithm. We can see that both SCIPR-gdy and SCIPR-mnn do indeed converge to a local optimum within the first few iterations. Fast convergence within the first few iterations is expected for Iterative Closest Points-based algorithms [28]. This supports our choice to run the SCIPR methods for 5 iterations in the experiments we present in our work. (PDF) [file pcbi.1007939.s009.pdf]

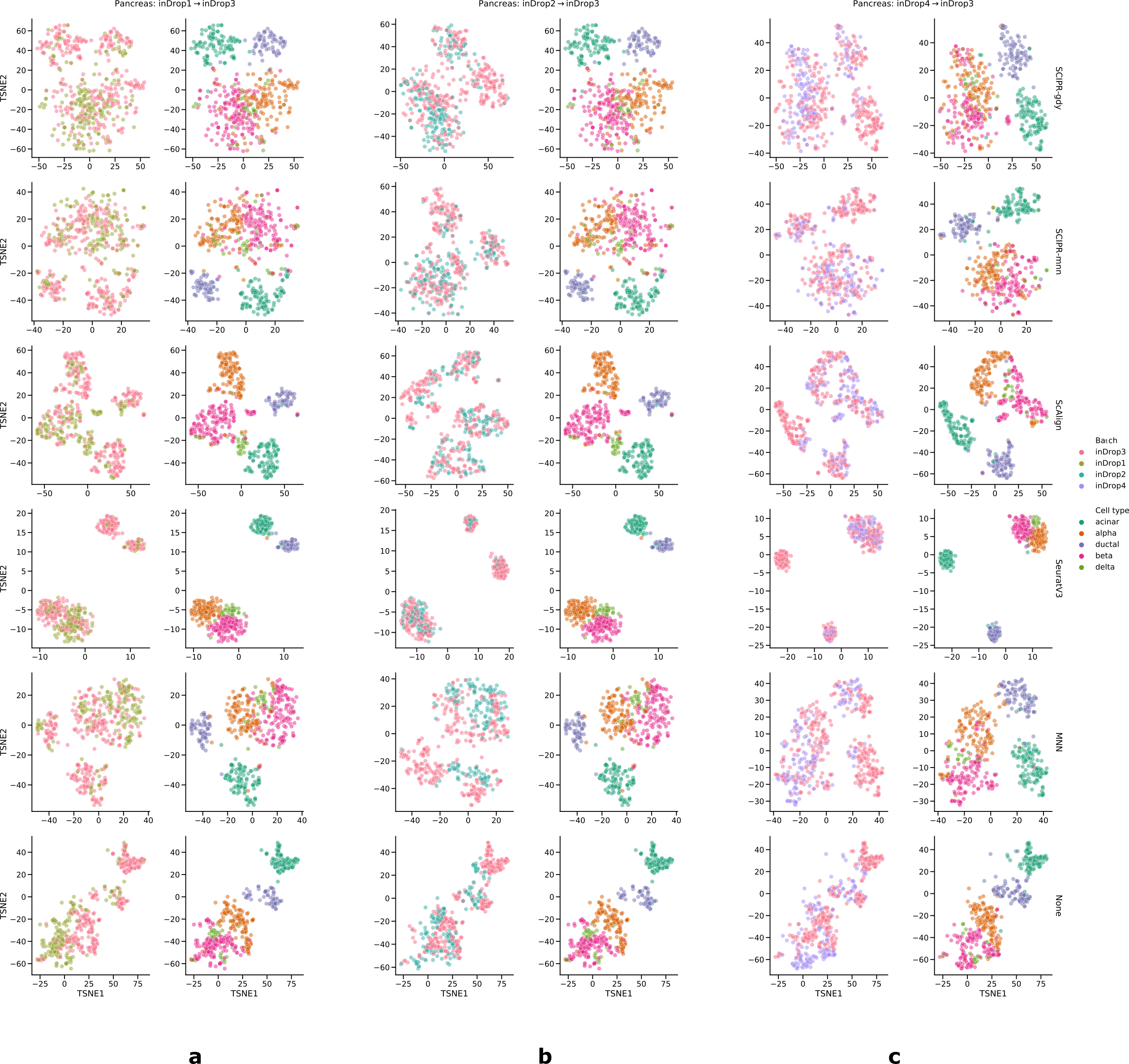

Supplement: S10 Fig — Each row is a different alignment method (the bottom row,“None”, is with no alignment). The columns are in three groups based on alignment task: the left two columns (a) pertain to aligning the inDrop1 batch onto the inDrop3 batch, the middle two columns (b) are for aligning the inDrop2 batch onto the inDrop3 batch, and the right two columns (c) are for aligning the inDrop4 batch onto the inDrop3 batch. The first, third, and fifth columns are colored by batch, and the second, fourth, and sixth columns are colored by cell type. (PNG) [file pcbi.1007939.s010.png]

SCI-PR-mnn

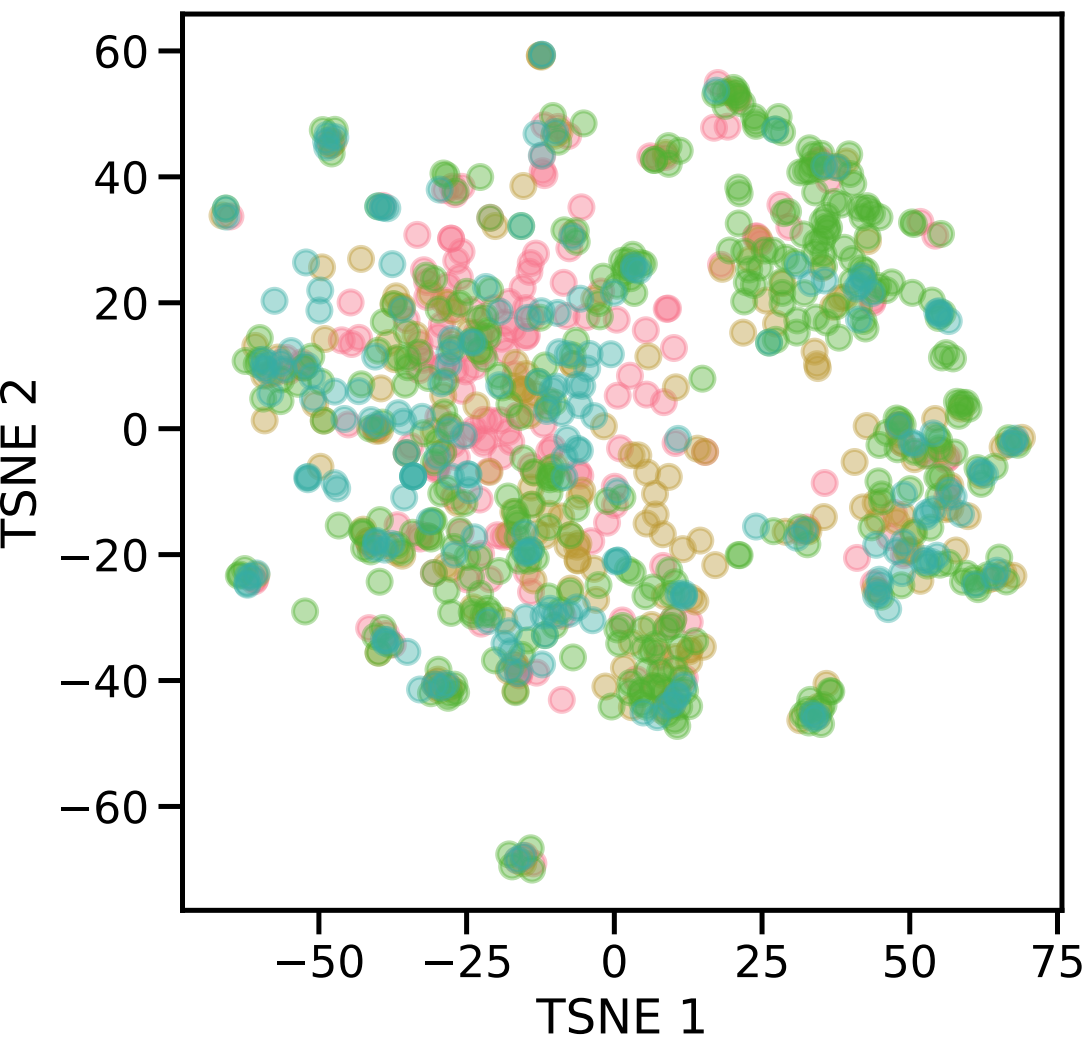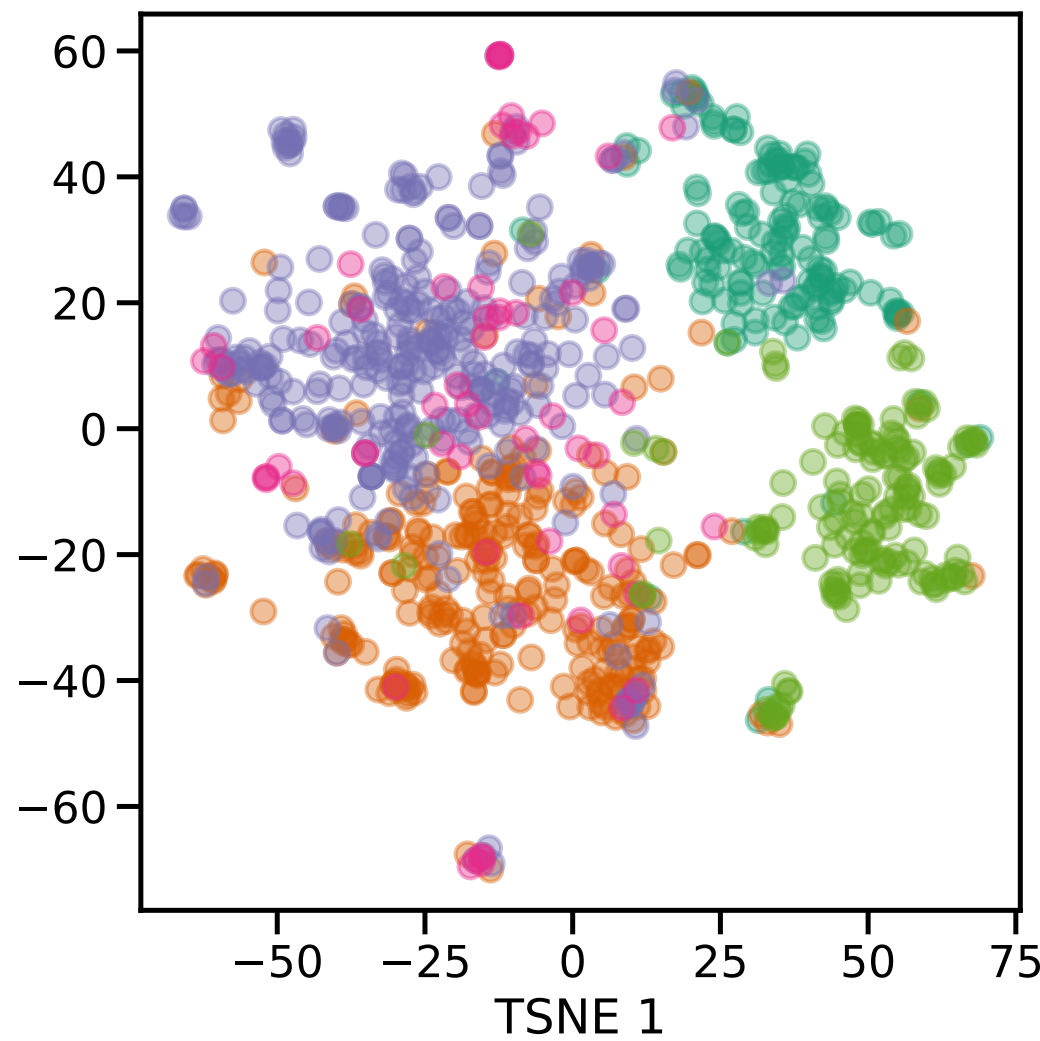

SCI-PR-gdy

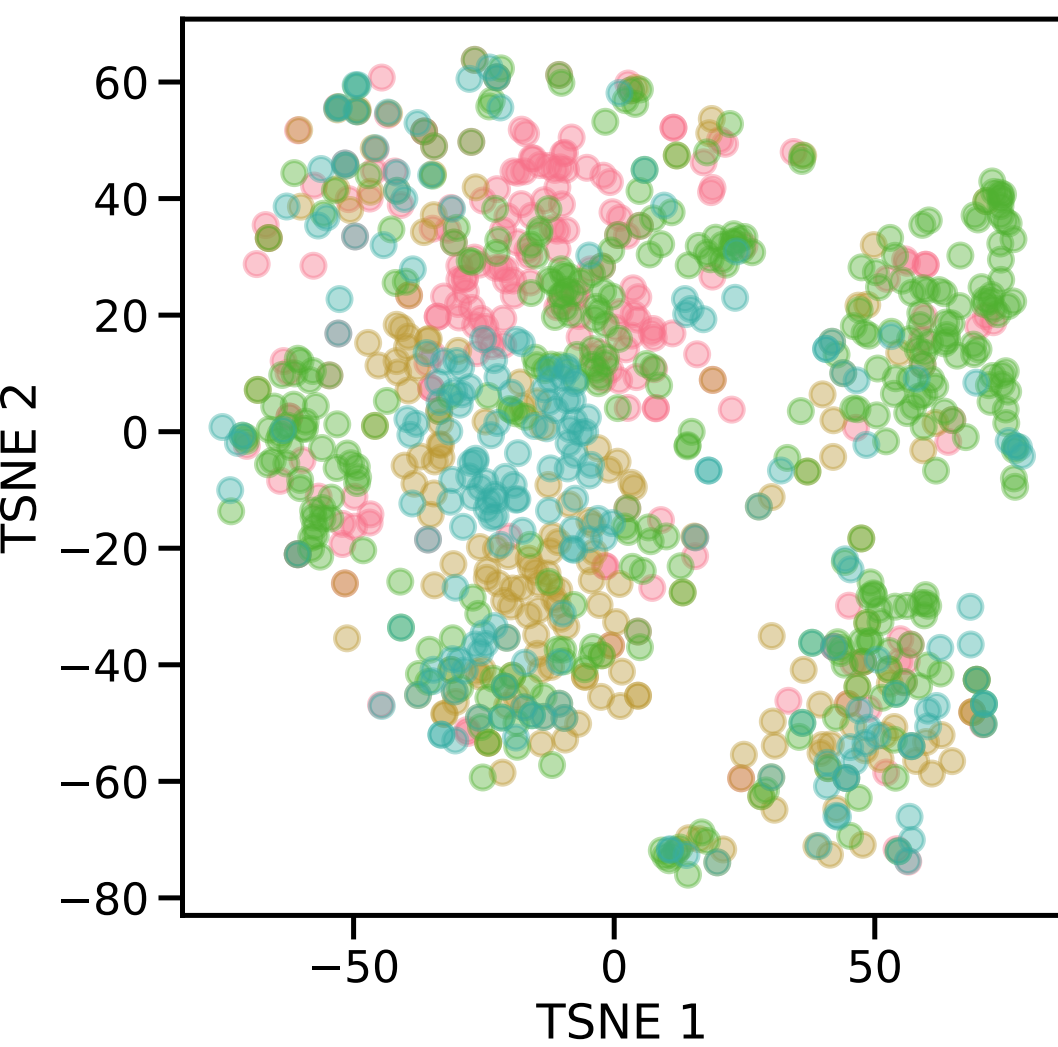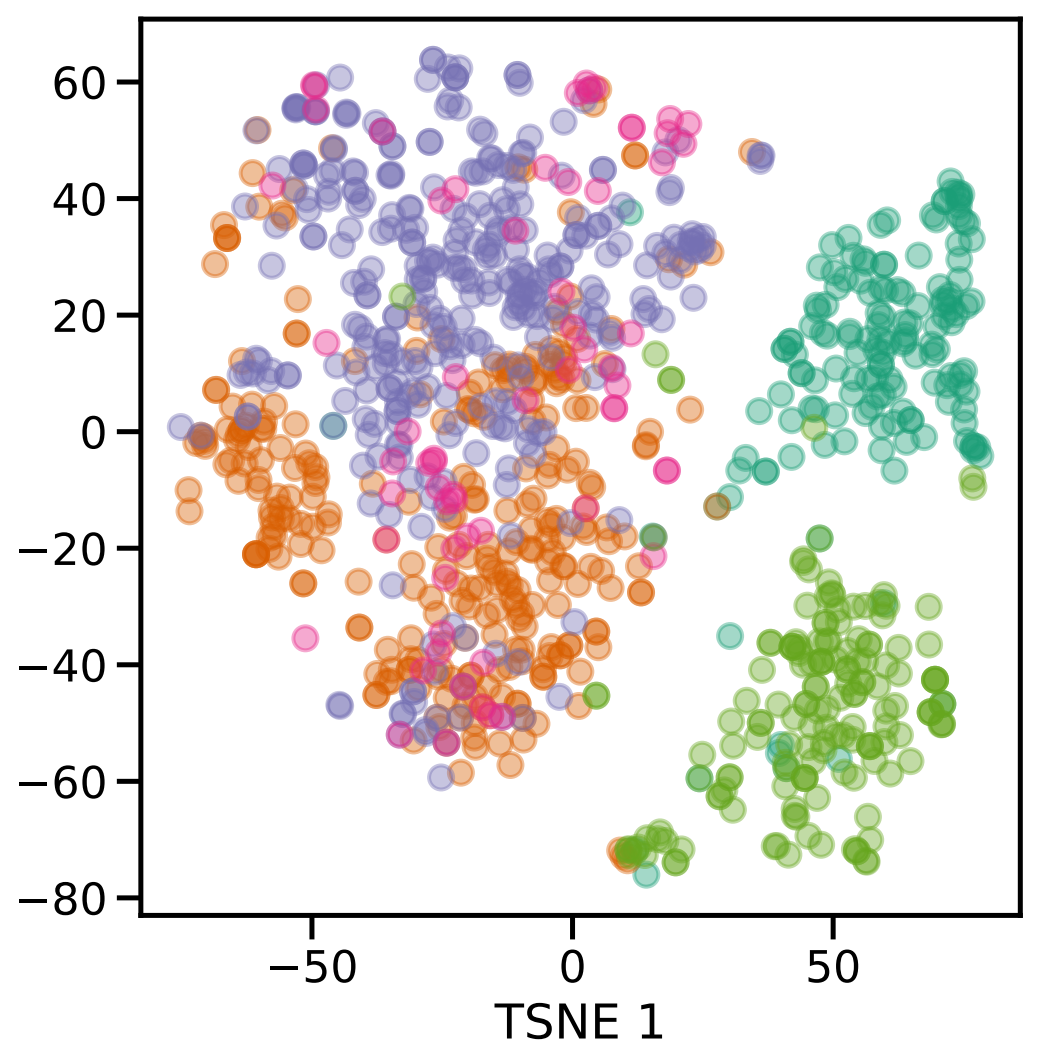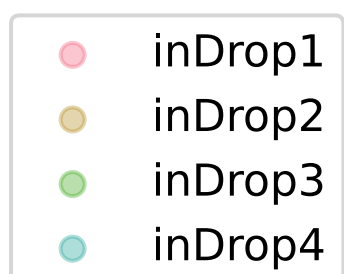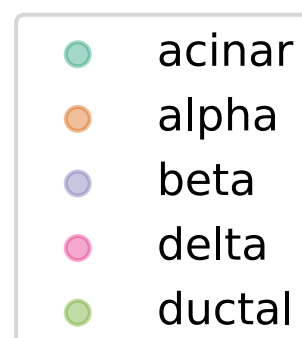

Supplement: S11 Fig — To see how SCIPR can be used to align multiple batches to a reference batch, we aligned each of the inDrop1, inDrop2, and inDrop4 batches to the inDrop3 batch (the largest batch) in the Pancreas dataset. These alignments were done independently, as pairwise alignments, and visualized together in the figure. In the subplots on the left, each point (cells) is colored by batch, and on the right they are colored by cell type. This straightforward multiple alignment strategy shows that it is possible to align many different batches to a single reference batch using SCIPR which results in coherent cell type representations while mixing the batches well. (PDF) [file pcbi.1007939.s011.pdf]

10x (v2) A  $\rightarrow$  10x (v2)10x (v2) B  $\rightarrow$  10x (v2)10x (v3)  $\rightarrow$  10x (v2)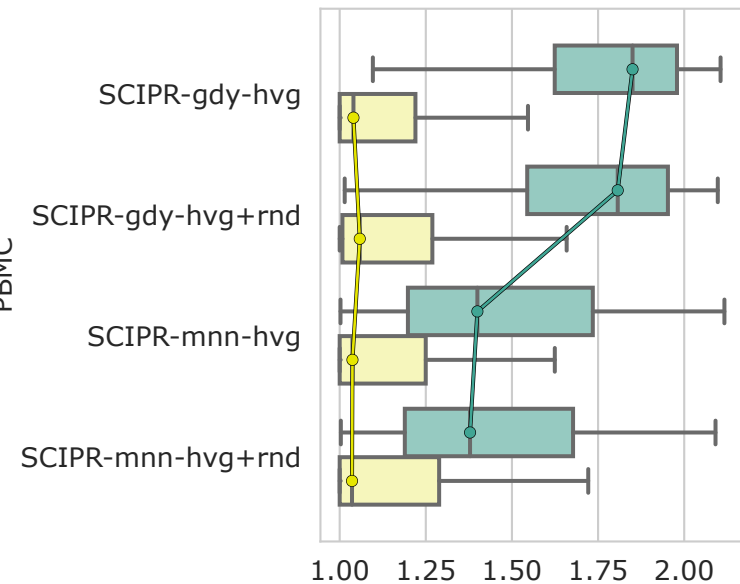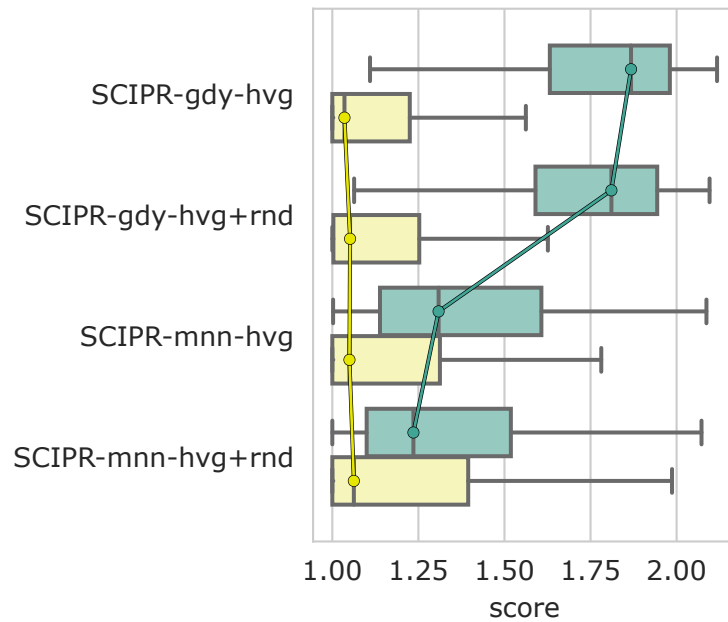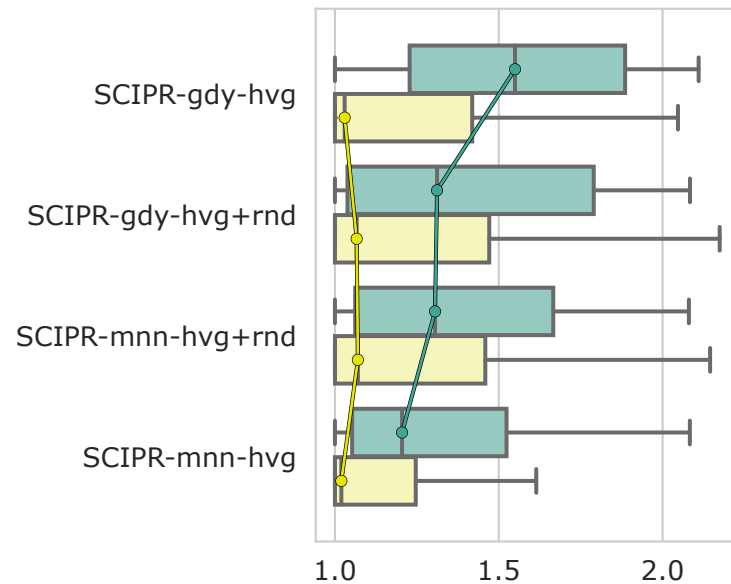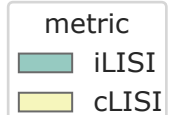

Supplement: S12 Fig — We compared using highly variable genes (as described in S1 Appendix “Data preprocessing and filtration”), versus using additional genes, for SCIPR models on the PBMC dataset (the largest dataset). Both our SCIPR-gdy and SCIPR-mnn models were run with either just the highly variable genes (“-hvg” suffix) (there are 1466 in the PBMC dataset) or with the highly variable genes and an equal number of randomly selected other genes (“-hvg+rnd” suffix). The three subplots correspond to the three different alignment tasks (aligning a source batch to a target batch) within the PBMC dataset. These quantitative scores show that SCIPR still performs well, and is robust to the inclusion of even more genes that are not necessarily the most informative genes. Box plot computation and ordering of methods in each subplot is determined in the same fashion as in S3 Fig. (PDF) [file pcbi.1007939.s012.pdf]
