# Supplementary material for: Iterative point set registration for aligning scRNA-seq data
Source: PLoS Comput Biol. 2020 Oct 27;16(10):e1007939. doi: 10.1371/journal.pcbi.1007939 (PMC7647120; doi:10.1371/journal.pcbi.1007939)
Supplement: S1 Table — Cell type and batch distributions for three scRNA-seq datasets we use for evaluation. Each row pertains to a batch, each column pertains to a cell type, and each value is the number of cells for each row and column combination. Numbers here are after our preprocessing described in S1 Appendix section “Data preprocessing and filtration”. The largest batch in each dataset is bolded, which we use as our reference “target” batch in our alignment tasks. (PDF) [file pcbi.1007939.s013.pdf]

Cell type<br>Batch	H1975	H2228	HCC827	total
**10x**	310	312	273	895
CELseq2	103	67	70	240
Dropseq	79	65	66	210

(a) CellBench dataset (GEO: GSE118767)

Cell type<br>Batch	acinar	alpha	beta	delta	ductal	total
inDrop1	58	160	519	110	93	940
inDrop2	87	326	221	58	146	838
**inDrop3**	468	438	312	26	244	1488
inDrop4	59	184	351	71	169	834

(b) Pancreas dataset (GEO: GSE84133)

Cell type<br>Batch	B cell	CD4+ T cell	Cytotoxic T cell	total
10x Chrom. (v2) A	287	550	1174	2011
10x Chrom. (v2) B	388	905	953	2246
10x Chrom. (v3)	346	960	962	2268
**10x Chrom. (v2)**	861	955	694	2510

(c) PBMC dataset (GEO: GSE132044)

Table S1
