# Supplementary material for: Iterative point set registration for aligning scRNA-seq data
Source: PLoS Comput Biol. 2020 Oct 27;16(10):e1007939. doi: 10.1371/journal.pcbi.1007939 (PMC7647120; doi:10.1371/journal.pcbi.1007939)
Supplement: S2 Table — (PDF) [file pcbi.1007939.s014.pdf]

GO term	Corrected p-val	intersection	reference	enquiry	background
COTRANSLATIONAL PROTEIN TARGETING TO MEMBRANE	0.000000e+00*	76	96	500	10518
CELLULAR AMIDE METABOLIC PROCESS	0.000000e+00*	122	743	500	10518
ORGANIC CYCLIC COMPOUND CATABOLIC PROCESS	0.000000e+00*	99	451	500	10518
TRANSLATIONAL INITIATION	9.709302e-10	91	174	500	10518
INTRACELLULAR PROTEIN TRANSPORT	3.188832e-09	107	885	500	10518
DEFENSE RESPONSE	3.188832e-09	92	906	500	10518
PROTEIN TARGETING	3.188832e-09	91	340	500	10518
LYMPHOCYTE ACTIVATION	3.188832e-09	72	473	500	10518
T CELL ACTIVATION	3.188832e-09	51	317	500	10518
ADAPTIVE IMMUNE RESPONSE	3.188832e-09	44	273	500	10518
INTRACELLULAR TRANSPORT	3.188832e-09	123	1306	500	10518
PEPTIDE BIOSYNTHETIC PROCESS	3.188832e-09	114	553	500	10518
REGULATION OF CELL ACTIVATION	3.411453e-09	54	356	500	10518
RIBOSOME BIOGENESIS	3.411453e-09	43	252	500	10518
CELLULAR MACROMOLECULE LOCALIZATION	3.411453e-09	125	1331	500	10518
POSITIVE REGULATION OF IMMUNE SYSTEM PROCESS	3.411453e-09	76	701	500	10518
PROTEIN LOCALIZATION TO MEMBRANE	3.411453e-09	83	418	500	10518
CELLULAR MACROMOLECULE CATABOLIC PROCESS	3.411453e-09	111	887	500	10518
REGULATION OF T CELL ACTIVATION	3.411453e-09	37	213	500	10518
POSITIVE REGULATION OF CELL ACTIVATION	3.411453e-09	41	231	500	10518

Table S2: Gene enrichment analysis of Differential Expression results. Differential expression results are from testing for differentially expressed genes in CD4+ T cells in the "10x Chromium (v2) A" batch of the PBMC dataset (CD4+ T cells vs. all other cell types). GO terms are from the "Biological Process" domain.

* The p-value was non-zero but very small and within floating point precision equal to zero.
