# Supplementary material for: Iterative point set registration for aligning scRNA-seq data
Source: PLoS Comput Biol. 2020 Oct 27;16(10):e1007939. doi: 10.1371/journal.pcbi.1007939 (PMC7647120; doi:10.1371/journal.pcbi.1007939)
Supplement: S3 Table — (PDF) [file pcbi.1007939.s015.pdf]

GO term	Corrected p-val	intersection	reference	enquiry	background
DEFENSE RESPONSE	9.743408e-09	95	906	500	10518
REGULATION OF IMMUNE RESPONSE	9.743408e-09	75	676	500	10518
HUMORAL IMMUNE RESPONSE	9.743408e-09	26	95	500	10518
LYMPHOCYTE ACTIVATION	9.743408e-09	61	473	500	10518
REGULATION OF CELL ACTIVATION	2.599720e-08	49	356	500	10518
REGULATION OF IMMUNE SYSTEM PROCESS	2.599720e-08	99	981	500	10518
B CELL MEDIATED IMMUNITY	2.599720e-08	22	80	500	10518
POSITIVE REGULATION OF IMMUNE SYSTEM PROCESS	3.605960e-08	74	701	500	10518
ADAPTIVE IMMUNE RESPONSE	3.605960e-08	41	273	500	10518
INNATE IMMUNE RESPONSE	4.770427e-08	65	580	500	10518
REGULATION OF LYMPHOCYTE ACTIVATION	5.137285e-08	43	299	500	10518
HUMORAL IMMUNE RESPONSE MEDIATED BY CIRCULATIN...	1.173927e-07	13	29	500	10518
B CELL RECEPTOR SIGNALING PATHWAY	4.539188e-07	17	58	500	10518
IMMUNE RESPONSE REGULATING CELL SURFACE RECEPT...	7.247366e-07	42	318	500	10518
LYMPHOCYTE MEDIATED IMMUNITY	8.827507e-07	29	171	500	10518
B CELL ACTIVATION	1.007833e-06	30	183	500	10518
CELL ACTIVATION	2.547541e-06	83	922	500	10518
IMMUNE EFFECTOR PROCESS	3.600901e-06	76	822	500	10518
REGULATION OF B CELL ACTIVATION	3.600901e-06	21	102	500	10518
LEUKOCYTE MIGRATION	3.600901e-06	33	229	500	10518

Table S3: Gene enrichment analysis of model weights from SCIPR-mnn. Model weights are fit to align cells (unsupervised) from the “10x Chromium (v2) A” batch to the “10x Chromium (v2)” batch.
