# Supplementary material for: Iterative point set registration for aligning scRNA-seq data
Source: PLoS Comput Biol. 2020 Oct 27;16(10):e1007939. doi: 10.1371/journal.pcbi.1007939 (PMC7647120; doi:10.1371/journal.pcbi.1007939)
Supplement: S4 Table — (PDF) [file pcbi.1007939.s016.pdf]

GO term	Corrected p-val	intersection	reference	enquiry	background
INNATE IMMUNE RESPONSE	0.000010	61	580	500	10518
HUMORAL IMMUNE RESPONSE MEDIATED BY CIRCULATIN...	0.000010	12	29	500	10518
B CELL MEDIATED IMMUNITY	0.000010	19	80	500	10518
COMPLEMENT ACTIVATION	0.000013	12	31	500	10518
DEFENSE RESPONSE	0.000018	81	906	500	10518
HUMORAL IMMUNE RESPONSE	0.000018	20	95	500	10518
REGULATION OF IMMUNE SYSTEM PROCESS	0.000022	85	981	500	10518
REGULATION OF LYMPHOCYTE ACTIVATION	0.000022	38	299	500	10518
LYMPHOCYTE ACTIVATION	0.000022	51	473	500	10518
LYMPHOCYTE MEDIATED IMMUNITY	0.000023	27	171	500	10518
REGULATION OF CELL ACTIVATION	0.000027	42	356	500	10518
NEUTROPHIL MIGRATION	0.000084	14	54	500	10518
POSITIVE REGULATION OF IMMUNE SYSTEM PROCESS	0.000142	64	701	500	10518
RESPONSE TO BIOTIC STIMULUS	0.000361	52	539	500	10518
REGULATION OF IMMUNE RESPONSE	0.000361	61	676	500	10518
LEUKOCYTE CELL CELL ADHESION	0.000365	29	224	500	10518
DEFENSE RESPONSE TO BACTERIUM	0.000390	18	100	500	10518
PHAGOCYTOSIS RECOGNITION	0.000468	8	19	500	10518
GRANULOCYTE MIGRATION	0.000501	14	64	500	10518
POSITIVE REGULATION OF LYMPHOCYTE ACTIVATION	0.000501	27	205	500	10518

Table S4: Gene enrichment analysis of model weights from SCIPR-gdy. Model weights are fit to align cells (unsupervised) from the “10x Chromium (v2) A” batch to the “10x Chromium (v2)” batch.
