# Supplementary material for: Iterative point set registration for aligning scRNA-seq data
Source: PLoS Comput Biol. 2020 Oct 27;16(10):e1007939. doi: 10.1371/journal.pcbi.1007939 (PMC7647120; doi:10.1371/journal.pcbi.1007939)
Supplement: S5 Table — (PDF) [file pcbi.1007939.s017.pdf]

GO term	Corrected p-val	intersection	reference	enquiry	background
B CELL MEDIATED IMMUNITY	0.000004	20	80	500	10518
LYMPHOCYTE ACTIVATION	0.000005	54	473	500	10518
LYMPHOCYTE MEDIATED IMMUNITY	0.000019	28	171	500	10518
CELL ACTIVATION	0.000022	82	922	500	10518
RESPONSE TO BACTERIUM	0.000022	39	306	500	10518
REGULATION OF LYMPHOCYTE ACTIVATION	0.000029	38	299	500	10518
DEFENSE RESPONSE	0.000029	80	906	500	10518
B CELL ACTIVATION	0.000033	28	183	500	10518
RESPONSE TO BIOTIC STIMULUS	0.000042	55	539	500	10518
ADAPTIVE IMMUNE RESPONSE BASED ON SOMATIC RECO...	0.000048	27	177	500	10518
ADAPTIVE IMMUNE RESPONSE	0.000048	35	273	500	10518
INNATE IMMUNE RESPONSE	0.000061	57	580	500	10518
LEUKOCYTE PROLIFERATION	0.000061	27	181	500	10518
IMMUNOGLOBULIN PRODUCTION INVOLVED IN IMMUNOGL...	0.000099	12	40	500	10518
REGULATION OF CELL ACTIVATION	0.000155	40	356	500	10518
HUMORAL IMMUNE RESPONSE	0.000177	18	95	500	10518
POSITIVE REGULATION OF LYMPHOCYTE ACTIVATION	0.000177	28	205	500	10518
IMMUNE EFFECTOR PROCESS	0.000181	71	822	500	10518
LYMPHOCYTE ACTIVATION INVOLVED IN IMMUNE RESPONSE	0.000360	20	121	500	10518
B CELL ACTIVATION INVOLVED IN IMMUNE RESPONSE	0.000562	13	56	500	10518

Table S5: Gene enrichment analysis of highly variable genes. Enrichment of random subset of 500 of the 1466 highly variable genes in the PBMC dataset, versus a background of all genes assayed in the dataset. This experiment is a baseline, showing the enrichment present simply due to filtering to highly variable genes. The number of genes (500) was chosen to be the same as the number chosen in S7 Appendix and S8 Appendix to allow for fair comparison.
