# Supplementary material for: Iterative point set registration for aligning scRNA-seq data
Source: PLoS Comput Biol. 2020 Oct 27;16(10):e1007939. doi: 10.1371/journal.pcbi.1007939 (PMC7647120; doi:10.1371/journal.pcbi.1007939)
Supplement: S6 Table — (PDF) [file pcbi.1007939.s018.pdf]

Runtimes on task *Pancreas: inDrop1→inDrop3*			
Method	Runtime		
	hr	min	sec
ScAlign	1	14	43
SCIPR-mnn	1	7	46
SCIPR-gdy	0	21	59
SCIPR-mnn (gpu)	0	4	22
SeuratV3	0	2	34
MNN	0	1	10
SCIPR-gdy (gpu)	0	0	39

Table S6: Runtimes of alignment methods. We ran each alignment method on the same task: aligning cells from the inDrop1 batch (940 cells) to the inDrop3 batch (1488 cells) of the Pancreas dataset, using the 2629 most variable genes. The methods are sorted from top to bottom in order of longest to shortest runtime. All methods were run on the same exact machine, with 2 cores of an Intel Xeon CPU E5-2630 v4 @ 2.20GHz with 16Gb of memory. The SCIPR methods can automatically utilize a GPU if available to accelerate training. SCIPR (gpu) methods utilized an Nvidia GeForce GTX 1080 Ti GPU card.
