# Supplementary material for: Iterative point set registration for aligning scRNA-seq data
Source: PLoS Comput Biol. 2020 Oct 27;16(10):e1007939. doi: 10.1371/journal.pcbi.1007939 (PMC7647120; doi:10.1371/journal.pcbi.1007939)
Supplement: S1 Appendix — This appendix contains the details of supporting analysis methods, including how we preprocess and filter data, conduct differential expression analysis, discover top weighted genes in our SCIPR models, and conduct gene set enrichment analysis. It contains additional details on computing LISI scores and computing the final affine transformation from SCIPR models. It also includes details of our software pipeline and settings for related methods. (PDF) [file pcbi.1007939.s020.pdf]

# Supporting Methods for

# Iterative point set registration for aligning scRNA-seq data

**Amir Alavi**[1] and **Ziv Bar-Joseph**[1,2,*]

[1]Computational Biology Department

[2]Machine Learning Department

School of Computer Science, Carnegie Mellon University, Pittsburgh, Pennsylvania, United States of America

[*]Corresponding author: zivbj@cs.cmu.edu

## scRNA-seq alignment benchmarking software and data

We created a Python software pipeline to load datasets, specify alignment tasks between batches of these datasets, preprocess data, run different methods of scRNA-seq alignment (including our own), compute iLISI and cLISI alignment scores for each, and visualize the results. We provide this pipeline and data in a separate repository from our SCIPR code. It can be found in our repository at `https://github.com/AmirAlavi/sc-alignment-benchmarking`.

## Data preprocessing and filtration

For each dataset used in our experiments, we removed cells which had low detected genes and genes which were not detected in enough cells or had low number of reads. We did this for each batch within each dataset seperately. For the CellBench and Pancreas batches, we used cells which had a minimum of 1,800 non-zero genes (detected genes). For the batches in the PBMC dataset, we used a lower threshold of 250 genes, as that dataset had lower coverage in general. For filtering the genes, we required that each gene had at least 10 reads in the batch, and that it was detected (non-zero) in at least 5 cells in the batch (these same thresholds were used for all batches in all datasets). These gene filtering steps would result in different sets of genes being kept in each batch. Thus, for a dataset, the set of genes we keep is the intersection of those from each batch.

After this process of filtering out cells and genes based on read counts, for our SCIPR method and all other alignment methods we compared to, we further filtered the genes to the set of most highly variable genes for each dataset, across the batches. We used the method used by in the Seurat R package for scRNA-seq analysis [1], and implemented in the scanpy package for python[2] via the function `scanpy.pp.highly_variable_genes`.

## Software and settings for related methods

We compared our method to Mutual Nearest Neighbors (MNN) [3], SeuratV3 [4], and ScAlign [5]. We used the python implementation of MNN (mnnpy) [6] with default settings via the `mnnpy.mnn_correct` function. For SeuratV3, we followed the instructions for installation from `https://satijalab.org/seurat/` and used SeuratV3 with the default settings recommended in the "Standard Workflow" vignette. For ScAlign, we downloaded the code from the repository at `https://github.com/quon-titative-biology/scAlign`

and for easier integration into our python benchmarking framework, created python wrappers to call the same tensorflow functions in python as the R tensorflow code in their repository. We used the same default parameters as specified in their original code.

## Computing the final affine transformation at the end of SCIPR

Since our function class for $f_\theta$ is affine transformations, and the composition of affine transformations is itself an affine transformation, we can combine this chain of transformations into a single affine transformation. In this case, we represent $f_\theta(x) = W^T x + b$ as $f_\theta(x) = W'^T x$ where $W'$ is the augmented matrix that adds another dimension to include the translation term $b$ with the linear transformation in a single matrix $W'$. With this representation in hand, we can update our overall $f_\theta$ after each iteration by left multiplying the current $f_\theta$ by the latest $f_\theta$ learned in the current iteration. In the end after $T$ iterations of the algorithm, our final function is:

$$
\begin{aligned}
f_\theta &= f_\theta^{(T)} \circ f_\theta^{(T-1)} \circ \ldots \circ f_\theta^{(1)} \\
&= W'^{(T)} \cdot W'^{(T-1)} \cdot \ldots \cdot W'^{(1)}
\end{aligned}
$$

## Parameter settings for SCIPR experiments

In all of our experiments, we used the same default parameter settings for our SCIPR methods. We found that these settings were fairly robust to different input datasets, as we saw in our results, but users may want to experiment with different values to achieve the desired level of batch mixing:

- We first normalized the source and target batches by scaling each cell's gene expression vector to unit norm, as is also done in the MNN method [3]
- We ran the algorithm for 5 iterations, as we find fast convergence during the first few iterations (S9 Fig). This fast early convergence behavior is also reported in the original iterative closest point publication [7]. One could run the algorithm for even longer if desired, but the decrease in mean distances between corresponding cells is usually small after the first few iterations.
- We ran the gradient descent to the learn the affine transform at each iteration for 1000 steps

- We used a learning rate of 1e-3 for the gradient descent
- If MNN was used for the matching algorithm, we used 10 neighbors for computing mutual nearest neigbhors
- If our greedy algorithm, S1 Algorithm, was used for the matching, we used parameters $\alpha = 0.5$ and $\beta = 2$

## Computing iLISI and cLISI scores

As described in the Methods section 4.8, we use the local inverse Simpson's Index (LISI) metric [8] to evaluate our alignments, which measures the amount of diversity within a small neighborhood around each point in a dataset, with respect to a particular label. A good alignment will mix the batches well, and result in high diversity with respect to the batch label in local neighborhoods. On the other hand, a good alignment will simultaneously preserve biological signal (cellular state), and result in low diversity with respect to the cell type label in local neighborhoods. LISI is computed on a per-cell (per data point) basis as in the expression below:

$$LISI(x_i) = \frac{1}{\sum_{y \in Y} p(y|x_i)^2} \tag{S1}$$

Where $x_i \in \{x_1, ..., x_N\}$ is the expression vector of the $i$-th cell in the dataset of size $N$ and $Y$ is the set of unique values of the covariate with respect to which we are computing LISI. For example, when evaluating the integration LISI (iLISI), then $Y$ could be {10x, CELseq2, Dropseq, ...}. If we are evaluating cell-type LISI (cLISI) on the other hand, then $Y$ could be {Cytotoxic T Cell, CD4+ T Cell, B cell, ...}

The function $p(y|x_i)$ is a "relative abundance" of the covariate label $y$ in a local neighborhood around the point $x_i$. As in [8], we use Gaussian kernel-based distributions of neighborhoods, and use the same fixed perplexity of 30. Our implementation is a direct translation of the R implementation in [8] to Python.

Intuitively, the summation is capturing the expected number of cells needed to be sampled before two are drawn with the same covariate label. If all the cells in the local neighborhood have the same label, then the expression will be 1 (low diversity). If all cells can have one of two labels with equal probability, then the expression will be 2 (high diversity). In this way, LISI measures the effective number of batches in a local neighborhood [8].

## Differential expression analysis

To conduct the differential expression analysis on the PBMC dataset presented in S2 Table, we did one-vs-rest tests of each cell type vs the other cell types present in a batch. The statistical test used was a non-parameteric Wilcoxon rank sum test, as is used in the latest version of the Seurat package [4]. We used the implementation available in the diffxpy python package for differential gene expression analysis of scRNA-seq data [9] via the function `diffxpy.api.test.rank_test`. After computing the adjusted p-value for each gene, via the Benjamini-Hochberg procedure for controlling false discover rate (FDR) [10], we then select the top 500 genes by adjusted p-value for enrichment analysis, as outlined below in appendix section "Gene set enrichment analysis".

## Selecting top genes from SCIPR models for enrichment analysis

After fitting a SCIPR model to align a source batch onto a given target batch, we can then analyze the model's parameters to determine which genes it is giving more weight to in its affine transformation. The model's weights are a matrix $W \in \mathbb{R}^{d \times d}$, where $d$ is the number of genes. The rows of the matrix correspond to the input genes, and the columns correspond to the output genes. The dimensions of the matrix are the same, as SCIPR is a transformation from $R^d$ to $R^d$, i.e. the input genes and the output genes are the same. We determine the importance of each input gene by first taking the absolute value of this matrix, then dividing the diagonal elements by the the sum in each column. This effectively places in each diagonal a normalized measure of the amount of influence that input gene had on computing the output of the transformation, relative to the other input genes. We then select the top 500 of these diagonal elements (where each diagonal element corresponds to a gene) for enrichment analysis as in appendix section "Gene set enrichment analysis".

## Gene set enrichment analysis

Given a set of genes of interest (e.g. either top differentially expressed genes as in appendix section "Differential expression analysis" or most highly weighted genes from the SCIPR model as in appendix section "Selecting top genes from SCIPR models for enrichment analysis"), we would like to compare them to annotated sets of genes to see if there is significant overlap, or enrichment. We compare our sets of genes to sets in the "Biological Process" domain of the Gene Ontology (GO) [11, 12]. We conducted the hy-

pergeometric test for over-representation to quantify the significance of overlap between the gene sets and used the Benjamini-Hochberg procedure as above [10], using the implementation in the diffxpy package [9] via the function `diffxpy.api.enrich.test`. We specified the background set to be the full set of genes after the read count filtration outlined in appendix section “Data preprocessing and filtration”. In addition, we specified that the annotated gene sets from GO first be filtered to only include genes that exist in our background set.

# References

1. Satija R, Farrell JA, Gennert D, Schier AF, Regev A. Spatial reconstruction of single-cell gene expression data. Nature biotechnology. 2015;33(5):495–502.

2. Wolf FA, Angerer P, Theis FJ. SCANPY: large-scale single-cell gene expression data analysis. Genome biology. 2018;19(1):15.

3. Haghverdi L, Lun AT, Morgan MD, Marioni JC. Batch effects in single-cell RNA-sequencing data are corrected by matching mutual nearest neighbors. Nature biotechnology. 2018;36(5):421–427.

4. Stuart T, Butler A, Hoffman P, Hafemeister C, Papalexi E, Mauck III WM, et al. Comprehensive integration of single-cell data. Cell. 2019;177(7):1888–1902.

5. Johansen N, Quon G. scAlign: a tool for alignment, integration, and rare cell identification from scRNA-seq data. Genome biology. 2019;20(1):1–21.

6. Kang C. An implementation of MNN (Mutual Nearest Neighbors) correct in python; 2019. `https://github.com/chriscainx/mnnpy`.

7. Besl P, McKay ND. A method for registration of 3-D shapes. IEEE Transactions on Pattern Analysis and Machine Intelligence. 1992;14(2):239–256.

8. Korsunsky I, Millard N, Fan J, Slowikowski K, Zhang F, Wei K, et al. Fast, sensitive and accurate integration of single-cell data with Harmony. Nature methods. 2019; p. 1–8.

9. Fischer DS, Hölzlwimmer F, Theis FJ. Fast and scalable differential expression analysis on single-cell RNA-seq data; 2020. `https://github.com/theislab/diffxpy`.

10. Benjamini Y, Hochberg Y. Controlling the false discovery rate: a practical and powerful approach to multiple testing. Journal of the Royal statistical society: series B (Methodological). 1995;57(1):289–300.

11. Ashburner M, Ball CA, Blake JA, Botstein D, Butler H, Cherry JM, et al. Gene ontology: tool for the unification of biology. Nature genetics. 2000;25(1):25–29.

12. Consortium GO. The gene ontology resource: 20 years and still GOing strong. Nucleic acids research. 2019;47(D1):D330–D338.
