# Supplementary material for: Iterative point set registration for aligning scRNA-seq data
Source: PLoS Comput Biol. 2020 Oct 27;16(10):e1007939. doi: 10.1371/journal.pcbi.1007939 (PMC7647120; doi:10.1371/journal.pcbi.1007939)
Supplement: S1 Algorithm — (PDF) [file pcbi.1007939.s021.pdf]

Algorithm S1: Greedy pair assignment algorithm. It takes as input the distance matrix $\boldsymbol{D}$ (S1 Fig) and algorithm hyperparameters α and β and returns the set of pairings S between the source batch of cells A and the target batch of cells B. The rows of the matrix $\boldsymbol{D}$ correspond to cells in A, the columns correspond to cells in B, and the value at D_{ij} is the distance between cells $d(A_i, B_j)$ where $d(\cdot,\cdot)$ is the euclidean distance function. S is a set of tuples of indexers into sets A and B which will be chosen by the algorithm: $S \subseteq \{(i,j) \mid 1 \leq i \leq |A|,\ 1 \leq j \leq |B|\}$. The *countOccurences*$(j, S)$ function below counts the number of times the jth target cell appears in the set of pairings S.

Input: $\boldsymbol{D} \leftarrow$ distance matrix, $\alpha \leftarrow$ source set match threshold, $\beta \leftarrow$ target set node match limit

Result: $S \equiv$ pair set

$S \leftarrow \{\}$;

$P \leftarrow$ sortElementsAscending($\boldsymbol{D}$);

foreach D_{ij} *in* P **do**

  if $i \notin S$ *and countOccurences(j, S)* $\leq \beta$ **then** `/* assign pair if` i `hasn't been matched yet, and` j `hasn't been matched more than` β `times */`

    $S \leftarrow S \cup \{(i,j)\}$;

  end

  if $\frac{|S|}{|A|} \geq \alpha$ **then** `/* stop if more than` α `A cells have been assigned */`

    break;

  end

end
