# Supplementary material for: Iterative point set registration for aligning scRNA-seq data
Source: PLoS Comput Biol. 2020 Oct 27;16(10):e1007939. doi: 10.1371/journal.pcbi.1007939 (PMC7647120; doi:10.1371/journal.pcbi.1007939)
Supplement: Attachment — Submitted filename: author_response_to_reviews.pdf [file pcbi.1007939.s022.pdf]

Author Response to Reviews for “Iterative point set registration for aligning scRNA-seq data”

Amir Alavi and Ziv Bar-Joseph

Dear Dr. Shi-Jie Chen and Dr. Weixiong Zhang,

First, we would like to thank the editors and reviewers for their thoughtful critiques and feedback. We appreciate the care with which they have communicated their concerns, and we have taken this opportunity to apply their feedback to strengthen the work. Attached, please find a revised version of our manuscript PCOMPBIOL-D-20-00745 “Iterative point set registration for aligning scRNA-seq data”. In the revised version we have addressed all comments provided by the reviewers (revisions in red font).

Concerns raised by reviewers included proper selection of baseline methods and datasets, clarity of the writing, and figure quality. We have addressed these issues by performing additional analysis, clarifying the intended scope of the work, improving the clarity of the writing, and improving the quality of our figures.

For more specific concerns, we have attached a point-by-point response for individual reviewers below (using their original headings where provided). The reviewers' comments in black font are followed by our responses in blue font. Please note that any page numbers that we reference in this blue font refer to numbers in our revised manuscript, not the original submission.

Reviewer #1

Major

1. My primary concern regarding the benchmarking analyses presented in the first part of the methods has to do with choices in test datasets. First, the primary dataset that was analyzed (CellBench), while clearly useful for benchmarking, does not represent the usual complexity of single-cell RNA-seq datasets. More representative would be the pancreas dataset used. I would like to see the tSNE embeddings from the pancreas presented as well (similar to Figure 4).
We agree that the pancreas dataset is a more complicated dataset. As suggested, we added a new embedding plot for this data. See Supplementary Figure S10 (inserted at the end of the response as well) and the discussion in lines 127-129 of Section 2.2.

2. In addition, it concerns me that only highly variable genes were used during the analysis. While perhaps this was done to decrease the computational complexity, it will also decrease the usefulness of the transformation if only a small subset of genes can be used in downstream analyses. This limitation should be discussed and perhaps compared to a dataset filtered only on depth. In addition, I believe these filtering details should be included in the methods or results section as they are important to evaluating the method.

We agree that using more genes can improve the overall ability to perform alignment in gene space. While using only highly variable genes (HVGs) indeed decreases computational run time, our method can handle more genes. The main reason we have used such subset was to match the standard practice of current alignment methods so that we could fairly compare to them. For example, Seurat's guides and vignettes recommend the identification of HVGs before using their method ("Standard Workflow" vignette) (Stuart et al., 2019). However, our method could take as input much larger set of genes and even all genes.

Based on this comment we performed additional analysis using the PBMC dataset (the largest dataset we tested). In this analysis we combined the 1466 variable genes we initially used with 1466 additional, randomly selected, genes, (so using a total that is twice the number of genes we have used in the paper). Results are presented in Figure S12. As the figure shows, while we do see a decrease in performance when using additional (non variable) genes, results are still good for the larger set and can both mix the batches and separate cell types.

Given these results we revised the Discussion to mention this (lines 211-216) and have also added filtering details to Methods as requested (Methods Section 4.1 and further detailed in Appendix S2).

3. I am skeptical of the analysis presented in section 2.5. It is not obvious to me that you would expect the coefficients for batch effect correction to be relevant to the biological process. Would it not be more likely that the genes with the highest coefficients would be related to technical variation? (stress perhaps?)

Thank you to the reviewer for bringing up this point of confusion. The intuition may not be obvious to readers, so we have added a discussion of the reasoning behind the analysis to Section 2.5 (lines 163 - 166).

There are two sets of genes that may differ between the batches. The first are cell type specific genes and the second is the general (noise or stress as the reviewer mentions) genes. Note that the former would be different between cell types even in the same batch whereas the latter are unrelated (at least to a large extent) to cell type and so expected to differ similarly between *all* cells in the two batches. If the goal was simply to match one batch to the other the stress related genes may have indeed been selected as the most relevant (and so would result in the highest coefficients). However, our alignment strategy is different and attempts to align specific *pairs* of cells (one from each batch). Such alignment is focused much more strongly on cell type specific genes if it indeed aligns cells of the same type together, as we show it does. Our iterative process ensures that genes that are relevant to cell type (and so would be expected to show similar responses in the matched pairs) would be prioritized over the general genes which would lead to lower improvement for the *cell type specific* alignment.

We realize that this is just an hypothesis and so based on this and the comment below have done additional analysis which is described in details below.

4. Also on section 2.5, I don't believe proper controls were demonstrated in this analysis – we would expect highly variable genes to already be enriched in the relevant biological process. The authors compare the high coefficient genes selected by SCIPR to a background of all genes filtered by depth, including low variable genes that were not included in the SCIPR analysis. The authors should either use only the set of highly-variable genes as background, or provide a null analysis comparing a random subset of highly-variable genes to all genes filtered by depth. I believe these methods details should be included in the methods section and not in the supplement as they are crucial to interpretation.
This is an excellent point, and we thank the reviewer for bringing this to our attention. As suggested we have performed the comparison proposed by the reviewer. Specifically, to compare SCIPR based selection to other subsets we first select the highly variable genes in the PBMC dataset (1488 as determined by scanpy's pp.highly_variable_genes function, which uses Seurat's logic) as we have done for the SCIPR analysis. Next, we randomly sample 500 of these genes (the same number used in our SCIPR weights analysis and in the comparison DE enrichment analysis) and perform GO enrichment on these. As the reviewer mentions, we indeed see an enrichment of relevant categories for the variable gene set as well. However, it is also clear that the enrichment for the genes selected based on SCIPR weights is much stronger. For example, the term LYMPHOCYTE ACTIVATION has enrichment p-value of 9.743408e-09 based on our SCIPR-mnn weights and p-value of 5.0e-06 from the above random HVG analysis, the term ADAPTIVE IMMUNE RESPONSE has enrichment p-value of 3.605960e-08 based on our SCIPR-mnn weights and p-value of 4.8e-05 from the HVG analysis. See Table S5 (inserted at the end of the response as well) for complete results.

Minor

Line 32 – I believe some of the largest studies are combinatorial indexing based (split-seq/sci-rna-seq) rather than solely microfluidic
We thank the reviewer for bringing this to our attention, and have modified the language to also mention split-pool barcoding-based methods.

I found the iLISI/cLISI boxplots difficult to evaluate 'at a glance'. I think the visualization might be improved if overlaid by a line plot connecting the median values.
This is a great suggestion, and we have added the proposed line plots to all of our LISI visualizations to make quick evaluation easier for the reader.

Reviewer #2

Scientific issues

1. Since the authors developed two versions of SCIPR based on Mutual Nearest Neighbors (MNN) and greedy matching respectively, the authors are recommended to specify the concrete situations that each of the two algorithms is applicable.
As suggested we revised Discussion to address this point (lines 205 - 211). As we note there, we generally recommend the use of the MNN strategy which performs well and does not rely on hyper-parameters related to the expected matching between the datasets (thus, requires less assumptions). However, in cases where the user has prior belief or expectation on what portion of the cells should be matched between the two batches (for example, if s/he knows that some types are missing in one sample and so the match proportion is not expected to be high) then we recommend using the greedy algorithm, since such information can be incorporated using its hyperparameters and will thus lead to better results.

2. In Line 66-73, why can SCIPR (or ICP) enable the maintenance of semantics and generalization is not well addressed.
As suggested we have now added an explanation of how SCIPR maintains semantics and generalizes in that section (now lines 75 - 78): "A method based on ICP can maintain semantics because it operates in the input data space, meaning that if the input features are genes, then the output features will also be those same genes. Such methods can also generalize to unseen data because they are fitting a transformation function, which can then be applied to new batches."

3. To demonstrate the convergence rate of SCIPR, it is necessary to add a table or figure to illustrate the convergence rate on datasets of different sizes.
As suggested we have added Figure S9 (inserted below as well) depicting the convergence of the SCIPR algorithm on various datasets, and added a discussion on convergence to Methods Section 4.7 (lines 361 - 364). The values plotted are the mean distances between the selected pairs of points after each iteration of the algorithm. We can see that both SCIPR-gdy and SCIPR-mnn do indeed converge to a local optimum within the first few iterations, even on the largest dataset (PBMC). Fast convergence within the first few iterations is expected for Iterative Closest Points-based algorithms (Besl & McKay, 1992). In their work, Besl and McKay actually prove that ICP always monotonically converges to a local minimum with respect to the mean-square distance between the corresponding points based on the basic idea that 1) given corresponding points, finding the rigid transformation by minimizing a least squares objective will reduce the average distance and 2) the matching scheme to choose the corresponding points (closest point in their work) reduces the distance for each point individually. Here, we use least squares for affine transforms, and we use different matching algorithms. We forego a

formal proof of convergence, but we show empirically in Figure S9 that our algorithms do indeed converge to local optima very quickly, like in (Besl & McKay, 1992).

4. Because the final global transformation function is the composition of functions learnt in each iteration, it seems that the more iterations, the more parameters the global transformation function has. Will this lead to overfitting?
We may have not explained the function well. In fact, one of the benefits of using affine transformations as we do is that the number of parameters is fixed throughout the algorithm learning. Thus, while parameters are indeed updated in each iteration the number of parameters does not change. We now explicitly explain this in Methods Section 4.7 and Appendix S4. As we discuss there, the composition of multiple affine transformations (from each iteration) is simply a single affine transformation. Thus, the overall composed transformation has no more parameters than each of the individual step transformations. Note that this is an advantage of our method over non-linear neural network transformations which have many more parameters to fit.

5. In Figure 2, there are indrop1, 2, 3, and 4, but only indrop 1 and 3 are mentioned in Line 98-99. Then in Methods section (Line 206), the author only showed data information for indrop3 and 4. Also, the word "indrop" should be "inDrop".
For the description of 98-99 (now 103-104), this was simply meant as an example of describing an “alignment task” as we use the term in our paper. We wanted to familiarize the reader with the notation of {dataset}: {source batch} → {target batch}, which describes an alignment task of aligning cells of a chosen source batch onto the cells of a chosen target batch, within a dataset. We present all alignment tasks in Figure 2.
For the methods section in line 206 (now 239), we meant to give a brief description of the dataset for the reader, and we provide the full exact distribution of number of cells and cell types in all datasets in Table S1.
We have now capitalized “inDrop” correctly throughout the paper, thank you for bringing this to our attention.

6. In Table 1, Nine methods are presented. Why only four algorithms are selected for comparison? Besides, what does “none” represent in Figure 2 ~ 4?
The reason to show the many methods in Table 1 was to give someone new to the area of scRNA-seq alignment a view of the current methods at a glance. However, our paper is intended to be a ‘Methods’ paper and so its scope does not allow us to review or compare our approach to all methods available. We thus selected a subset of the most widely used methods for comparison. We have also tried to balance the comparisons to represent different strategies. These include methods based on vector-based corrections on top of a matching scheme (e.g. MNN and Seurat), and methods based on neural network embeddings (like ScAlign). There are a number of other papers whose purpose is to conduct a thorough analysis of alignment methods and alignment metrics (Chazarra-Gil et al., 2020; Luecken et al., 2020; Tian et al., 2019;) which we now mention to the reader on lines 66-67.

In Figures 2-4, “None” means no alignment method is applied to the data, and it is used in its original form. We have added this to the figure captions to make this clear.

7. In Figure 4, I can hardly tell which one is better between SCIPR-gdy and MNN.
We have now added a discussion of this in Results explaining that both perform well based on qualitative assessment of the embeddings in Figure 4, and further explain the tradeoff between the two and the overall choice between Greedy vs MNN in the Discussion (as mentioned in the response to Reviewer point 1 above).

8. The description of LISI, iLISI, and cLISI is less informative. For people not familiar with this score, it is hard to follow. Detailed algorithms are preferred to better explain these scores, as they are the most important validation method in this paper.
We thank the reviewer for bringing this up. In our original manuscript, we had not discussed the exact formula for how to compute LISI scores, as these are introduced in a previous paper from another group. We provided a summary in the Methods on lines 379 - 385 and cited that work to point the reader to the original source. Based on this comment we have now added Equation S1 along with a description of the steps in Appendix S6 “Computing iLISI and cLISI scores” to explain in detail how these scores are computed.

9. The paper showed integration of two scRNA-Seq datasets from different experiments/technologies, while in many cases multiple sample alignment is needed. How could SCIPR deal with such situation, align two datasets first and apply the third one, or align all three simutaneously? If it is the first situation, does the alignment sequence (e.g., 1+2+3 and 1+3+2) matter?
To address this comment we performed additional analysis in which we used SCIPR to perform multiple rather than pairwise alignment, to show that SCIPR can deal with this scenario. For this we used the four batches in the Pancreas dataset. One was used as the reference and the other three were (separately) aligned to it using SCIPR. We present the resultsin Figure S11 (inserted at the end of the response as well) (referenced on lines 107-108). As can be seen, SCIPR performs well for this multiple alignment tasks successfully mixing all batches while keeping cell types distinct.

10. In Section 2.5, the selected genes are selected according to their SCIPR coefficients. Why it is written that the parameters learnt can be used to identify CELL TYPE-SPECIFIC genes?

SCIPR alignment strategy attempts to align specific *pairs* of cells (one of each batch). When successful (i.e. when the resulting alignment indeed matches cells from the same type) such alignment is focused on cell type specific genes since these are expected to be the most highly expressed in the two matched cells. Our iterative process ensures that genes that are relevant to cell type (and so would be expected to show similar responses in the matched pairs) would be prioritized over other genes since this would lead to better *cell type specific* alignment. This is obviously just a hypothesis and so we performed the GO enrichment analysis to test it and as the results (Section 2.5) indicate it indeed seems to hold.

11. In section 2.5, I would prefer a toy case to show the comparison of DE results. Does SCIPR find more biological meaningful DE genes than other tools, such as Seurat? Stating the DE function and only using GO enrichment are insufficient to support the title of section 2.5.
Thank you for the suggestion. Following this comment and a comment from Reviewer #1 (point 4) we now performed additional analysis focused on the most variable rather than DE genes (Table S5, inserted below as well), while this indeed decreases computational complexity, our method can handle more genes.). For this set, we indeed see significant enrichment of relevant cell type genes. However, the enrichment achieved by SCIPR is much stronger (in terms of lower p-values) further supporting its use to identify cell type specific genes. With regards to Seurat, since it does not learn a model (e.g. a function that needs to be fit to data), we cannot inspect Seurat to see which genes it weighs more in its alignment. Also, conducting DE on the aligned output of Seurat and claiming biological significance would be problematic, as the DE analysis would be conducted on expression values generated by an algorithm meant to artificially integrate datasets, not on real experimentally observed expression.

12. As known, the number of GO terms overlapped with a gene set can be impacted by the size of the gene set and the size of the GO terms. Please present detailed information including the size of the gene set and the size of the GO term.
Thank you for requesting this. We have added columns to Supplementary Tables S2, S3, S4, and S5 which now include this overlap information.

13. It is necessary to add a comparison of efficiency between the methods.
As suggested we have now added a table of runtimes (Table S6) and discuss efficiency in the Methods Section 4.7 on lines 365-372. We find that SCIPR is indeed slower than some other methods due to its iterative learning procedure. However, the reduction in speed is compensated by the fact that unlike the faster methods such as Seurat and MNN, SCIPR actually learns a generalizable alignment function (which, for example, can be used to align data not seen or used for the learning). ScAlign, which also provides such function, has a runtime similar to SCIPR. SCIPR can also be accelerated with a GPU card, and the software will automatically search for and use the GPU if it is available. Table S9 shows that with a GPU, SCIPR-mnn and SCIPR-gdy can run on the same order of time (minutes) as MNN and Seurat, and in the case of SCIPR-gdy, can actually be faster.

14. A discussion should be added to explain the choice of parameters alpha, beta, and k.
We have now added a discussion in the Methods section on lines 319 - 324 (alpha, beta), 332 - 335 (k) to describe our choices for these hyperparameters further.

Presentation issues

1. A number of typos exist, for example, “non of the current methods …” in line 66.
We thank the reviewer for catching this typo, and we have fixed it.

2. The tables and figures can be further polished.
Thank you for bringing this up. We have now added panel labels to Figures 4 and 5, and have now added overlaying line plots to all of the LISI box plots to make them much easier to parse.

Reviewer #3

One of the major issues in scRNA-Seq is that the dropout rate is high (missing values of gene expression). It is unclear how the authors handled it. Did they use imputation? Some details should be provided.
Similar to other alignment methods we do not attempt to impute values and use the provided read counts ‘as is’. The main reason we (and all prior methods) do this is that dropouts are often correlated with batch-effects, which is exactly the point the alignment method is trying to address. Specifically, dropout rates for specific genes can vary depending on the scRNA-seq protocol used, and other technical factors. Thus, our alignment method serves as a type of imputation itself allowing us to assign much higher values to genes that are cell type specific and were dropped out (this is a potential outcome of the transformation function we learn).

The authors should discuss some limitations of their work. For example, the method uses an affine transforms, where non-linear properties are not considered.
We agree and have added the following to the Discussion (lines 223-226): ‘A potential drawback of SCIPR is the fact that it relies on affine transformations that do not handle non-linear differences between batches. While this leads to a reduction in the number of parameters that the method uses, and so reduces overfitting, it also means that the method cannot handle non-linear changes which may occur between different batches.’.

The writing of this paper can be improved. It is a little hard to follow. It may benefit from moving some text on problem formulation to Introduction or Results. Some discussions are sketchy. For example, in Figure 4, the Seurat's performance is also very good, but it was not mentioned.

We agree. Based on this and other comments we have made several changes to the paper. These are highlighted in red in the revised version. We also now explicitly mention the fact that Surat performs well on the data presented in Fig. 4 (line 127).

Figure quality needs some improvement. For example, A and B should be labelled in Fig. 5 (2).
This is a great suggestion. Figure panel labels have been added in Figures 4 and 5. In addition, we now added overlaying line plots to all of the LISI box plots to make them much easier to parse.

The language needs some edits, e.g.:
non of the current methods --> none of the current methods
We thank the reviewer for catching this typo, and we have fixed it.

New Figures

GO term	Corrected p-val	intersection	reference	enquiry	background
B CELL MEDIATED IMMUNITY	0.000004	20	80	500	10518
LYMPHOCYTE ACTIVATION	0.000005	54	473	500	10518
LYMPHOCYTE MEDIATED IMMUNITY	0.000019	28	171	500	10518
CELL ACTIVATION	0.000022	82	922	500	10518
RESPONSE TO BACTERIUM	0.000022	39	306	500	10518
REGULATION OF LYMPHOCYTE ACTIVATION	0.000029	38	299	500	10518
DEFENSE RESPONSE	0.000029	80	906	500	10518
B CELL ACTIVATION	0.000033	28	183	500	10518
RESPONSE TO BIOTIC STIMULUS	0.000042	55	539	500	10518
ADAPTIVE IMMUNE RESPONSE BASED ON SOMATIC RECO...	0.000048	27	177	500	10518
ADAPTIVE IMMUNE RESPONSE	0.000048	35	273	500	10518
INNATE IMMUNE RESPONSE	0.000061	57	580	500	10518
LEUKOCYTE PROLIFERATION	0.000061	27	181	500	10518
IMMUNOGLOBULIN PRODUCTION INVOLVED IN IMMUNOGL...	0.000099	12	40	500	10518
REGULATION OF CELL ACTIVATION	0.000155	40	356	500	10518
HUMORAL IMMUNE RESPONSE	0.000177	18	95	500	10518
POSITIVE REGULATION OF LYMPHOCYTE ACTIVATION	0.000177	28	205	500	10518
IMMUNE EFFECTOR PROCESS	0.000181	71	822	500	10518
LYMPHOCYTE ACTIVATION INVOLVED IN IMMUNE RESPONSE	0.000360	20	121	500	10518
B CELL ACTIVATION INVOLVED IN IMMUNE RESPONSE	0.000562	13	56	500	10518

Table S5: Gene enrichment analysis of highly variable genes. Enrichment of random subset of 500 of the 1466 highly variable genes in the PBMC dataset, versus a background of all genes assayed in the dataset. This experiment is a baseline, showing the enrichment present simply due to filtering to highly variable genes. The number of genes (500) was chosen to be the same as the number chosen in Appendix S7 and S8 to allow for fair comparison.

Runtimes on task *Pancreas: inDrop1→inDrop3*			
Method	Runtime		
	hr	min	sec
ScAlign	1	14	43
SCIPR-mnn	1	7	46
SCIPR-gdy	0	21	59
SCIPR-mnn (gpu)	0	4	22
SeuratV3	0	2	34
MNN	0	1	10
SCIPR-gdy (gpu)	0	0	39

Table S6: Runtimes of alignment methods. We ran each alignment method on the same task: aligning cells from the inDrop1 batch (940 cells) to the inDrop3 batch (1488 cells) of the Pancreas dataset, using the 2629 most variable genes. The methods are sorted from top to bottom in order of longest to shortest runtime. All methods were run on the same exact machine, with 2 cores of an Intel Xeon CPU E5-2630 v4 @ 2.20GHz with 16Gb of memory. The SCIPR methods can automatically utilize a GPU if available to accelerate training. SCIPR (gpu) methods utilized an Nvidia GeForce GTX 1080 Ti GPU card.

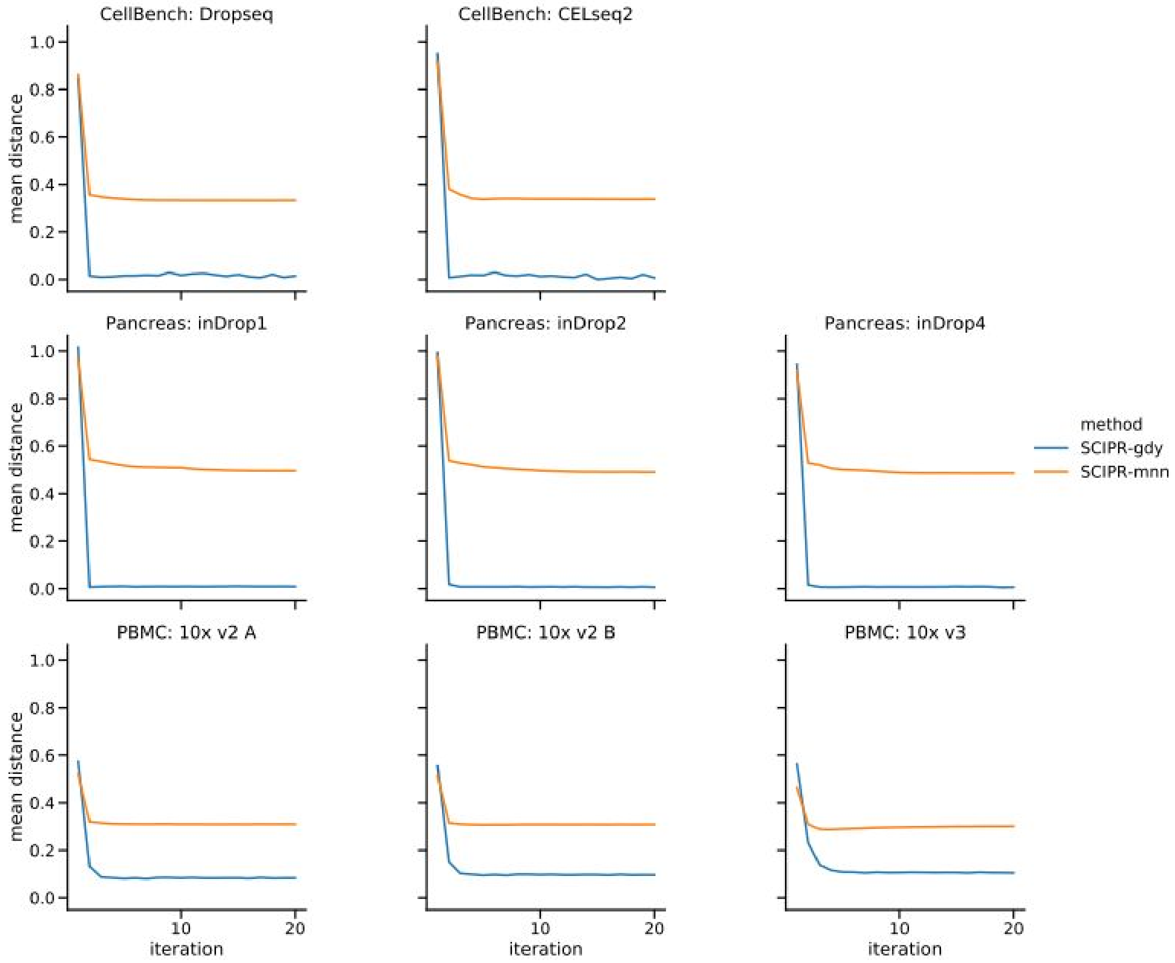

Figure S9: Convergence during fitting of SCIPR models. Each row of subplots are tasks from the same dataset, where each column uses a different source batch (all are aligned to the same largest reference batch, 10x for CellBench, inDrop3 for Pancreas, and 10x v2 for PBMC). The values plotted are the mean distances between the selected pairs of points after each iteration of the algorithm. We can see that both SCIPR-gdy and SCIPR-mnn do indeed converge to a local optimum within the first few iterations. Fast convergence within the first few iterations is expected for Iterative Closest Points-based algorithms [7]. This supports our choice to run the SCIPR methods for 5 iterations in the experiments we present in our work.

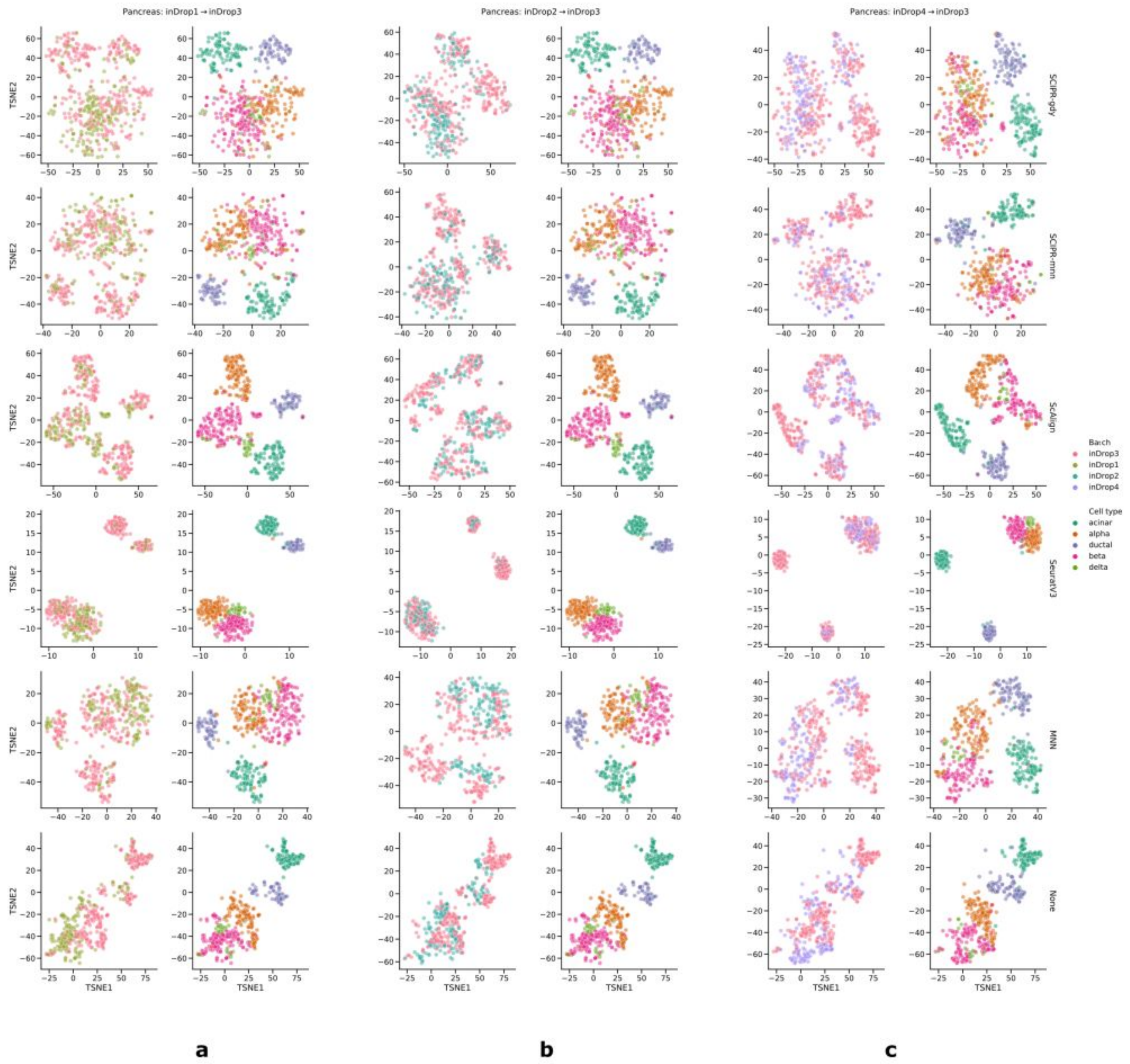

Figure S10: Embedding (t-SNE) visualization from alignment tasks on the Pancreas dataset using various alignment methods. Each row is a different alignment method (the bottom row, "None", is with no alignment). The columns are in three groups based on alignment task: the left two columns (a) pertain to aligning the inDrop1 batch onto the inDrop3 batch, the middle two columns (b) are for aligning the inDrop2 batch onto the inDrop3 batch, and the right two columns (c) are for aligning the inDrop4 batch onto the inDrop3 batch. The first, third, and fifth columns are colored by batch, and the second, fourth, and sixth columns are colored by cell type.

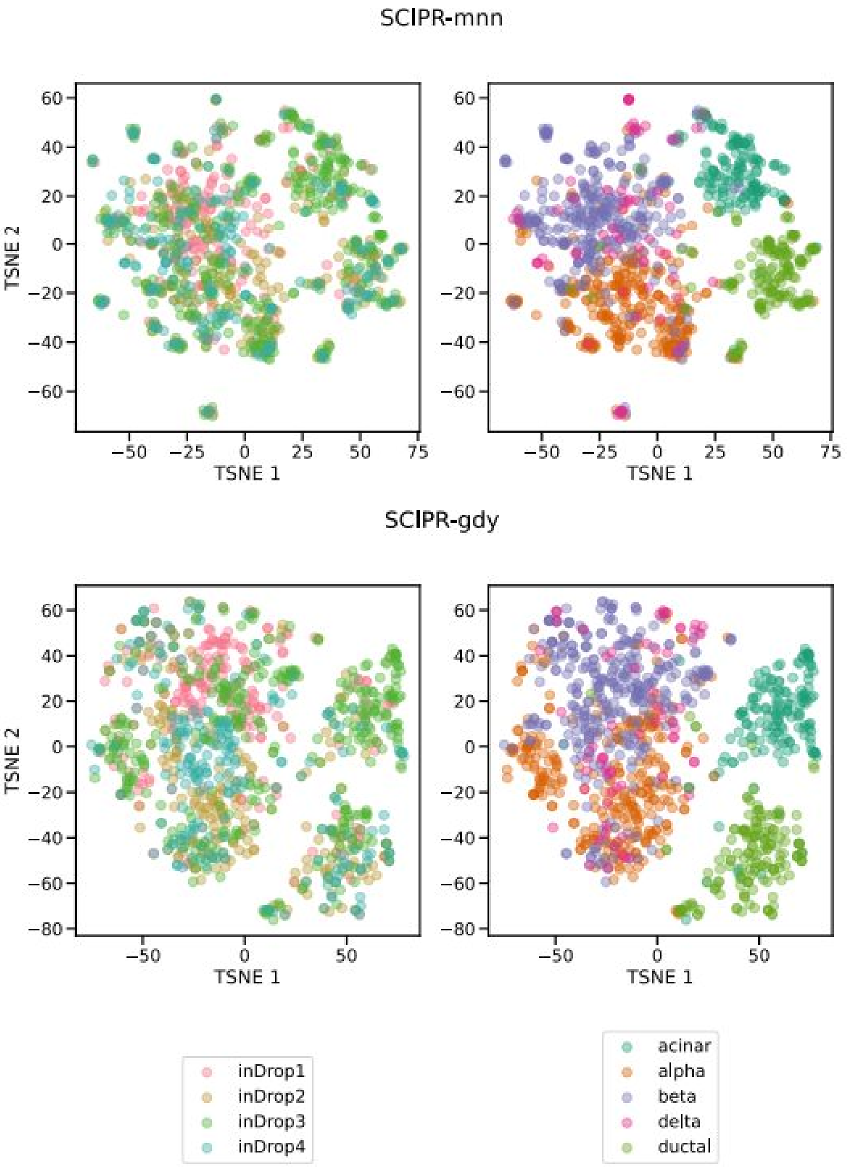

Figure S11: Example of aligning multiple batches to a reference batch using SCIPR. To see how SCIPR can be used to align multiple batches to a reference batch, we aligned each of the inDrop1, inDrop2, and inDrop4 batches to the inDrop3 batch (the largest batch) in the Pancreas dataset. These alignments were done independently, as pairwise alignments, and visualized together in the figure. In the subplots on the left, each point (cells) is colored by batch, and on the right they are colored by cell type. This straightforward multiple alignment strategy shows that it is possible to align many different batches to a single reference batch using SCIPR which results in coherent cell type representations while mixing the batches well.

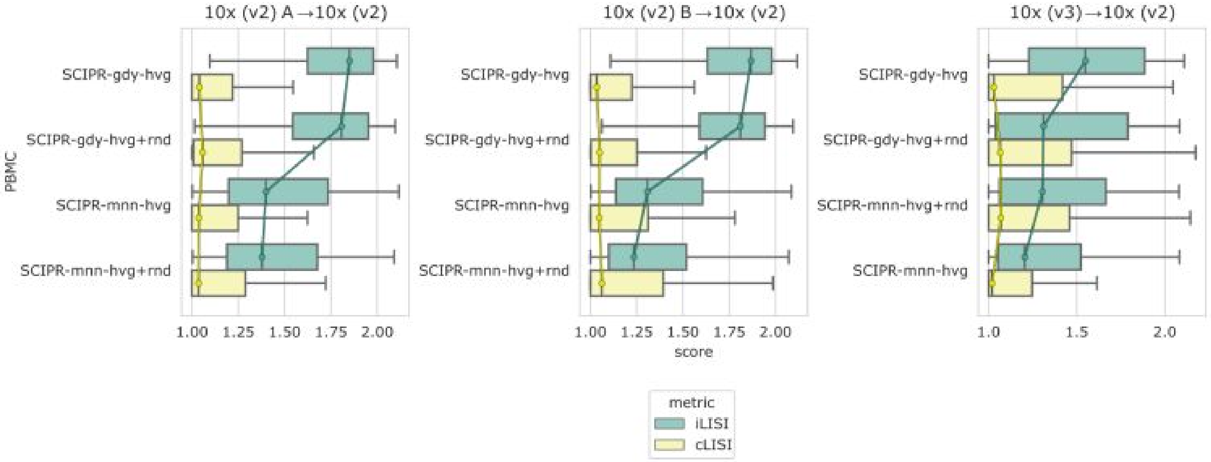

Figure S12: Comparison of using highly variable genes versus additional random genes in SCIPR. We compared using highly variable genes (as described in Appendix S2), versus using additional genes, for SCIPR models on the PBMC dataset (the largest dataset). Both our SCIPR-gdy and SCIPR-mnn models were run with either just the highly variable genes ("-hvg" suffix) (there are 1466 in the PBMC dataset) or with the highly variable genes and an equal number of randomly selected other genes ("-hvg+rnd" suffix). The three subplots correspond to the three different alignment tasks (aligning a source batch to a target batch) within the PBMC dataset. These quantitative scores show that SCIPR still performs well, and is robust to the inclusion of even more genes that are not necessarily the most informative genes. Box plot computation and ordering of methods in each subplot is determined in the same fashion as in Figure S3.

Bibliography

Besl, P., & Mckay, N. D. (1992). A method for registration of 3-D shapes. *IEEE Transactions on Pattern Analysis and Machine Intelligence*, *14*(2), 239–256. https://doi.org/10.1109/34.121791

Chazarra-Gil, R., Dongen, S. V., Kiselev, V. Y., & Hemberg, M. (2020). Flexible comparison of batch correction methods for single-cell RNA-seq using BatchBench. https://doi.org/10.1101/2020.05.22.111211

Luecken, M., Büttner, M., Chaichoompu, K., Danese, A., Interlandi, M., Mueller, M., … Theis, F. (2020). Benchmarking atlas-level data integration in single-cell genomics. https://doi.org/10.1101/2020.05.22.111161

Stuart, T., Butler, A., Hoffman, P., Hafemeister, C., Papalexi, E., Mauck, W. M., … Satija, R. (2019). Comprehensive Integration of Single-Cell Data. *Cell*, *177*(7). https://doi.org/10.1016/j.cell.2019.05.031

Tian, L., Dong, X., Freytag, S., Cao, K.-A. L., Su, S., Jalalabadi, A., … Ritchie, M. E. (2019). Benchmarking single cell RNA-sequencing analysis pipelines using mixture control experiments. *Nature Methods*, *16*(6), 479–487. https://doi.org/10.1038/s41592-019-0425-8
